# ZMAT1 acts as a tumor suppressor in pancreatic ductal adenocarcinoma by inducing SIRT3/p53 signaling pathway

**DOI:** 10.1186/s13046-022-02310-8

**Published:** 2022-04-07

**Authors:** Zuyi Ma, Zhenchong Li, Shujie Wang, Zixuan Zhou, Chunsheng Liu, Hongkai Zhuang, Qi Zhou, Shanzhou Huang, Chuanzhao Zhang, Baohua Hou

**Affiliations:** 1 Department of General Surgery, Guangdong Provincial People’s Hospital, Guangdong Academy of Medical Sciences, Guangzhou, 510080 China; 2 Shantou University of Medical College, Shantou, 515000 China; 3 South China University of Technology School of Medicine, Guangzhou, 51000 China; 4 The Second School of Clinical Medicine, Southern Medical University, Guangzhou, 510515 China; 5 Department of General Surgery, Hui Ya Hospital of The First Affiliated Hospital, Sun Yat-Sen University, Huizhou, 516081 China; 6 Department of Liver Surgery, The First Affiliated Hospital of Sun Yat-Sen University, Guangzhou, 510000 China

## Abstract

**Background:**

Pancreatic ductal adenocarcinoma (PDAC) is one of the deadliest cancer due to its highly aggressive phenotype and lack of effective biomarkers or treatment strategies. ZMAT1 belongs to the C2H2 type zinc finger family, but its biological function is rarely investigated, as well as its role in cancer development.

**Methods:**

Multiple bioinformatics analyses were used to evaluate ZMAT1 expression and potential role in PDAC. Intro and vivo studies were performed to assess the effects of ZMAT1 on PDAC cells growth. Furthermore, CHIP-seq and luciferase reporter assay was conducted to identify its specific regulatory mechanism in PDAC.

**Results:**

The current study identified the down-regulation of ZMAT1 and its associations with unfavorable clinicopathological characteristics and poor survival of PDAC. Further, we found overexpression of ZMAT1 inhibited pancreatic cancer cell proliferation by inducing p21, leading to impaired S/G2 cell cycle progression. Besides, over-expression of ZMAT1 led to decreased pancreatic cancer cell apoptosis. Mechanistically, ZMAT1 up-regulated p53 expression and inhibition of p53 abrogated the effect of ZMAT1 over-expression on pancreatic cancer cell, indicating the role of ZMAT1 in PDAC was dependent on p53. By performing CHIP-seq assay, we found ZMAT1 did not bind to P53 but bound to the promoter region of SIRT3, an upstream regulator for p53. Luciferase reporter assay showed transfection of ZMAT1 induced SIRT3 transcription, suggesting ZMAT1 was a transcriptional activator for SIRT3.

**Conclusion:**

Our findings indicated the role of ZMAT1-SIRT3-p53 signaling pathway during tumor growth, highlighting that ZMAT1 is a tumor suppressor and novel biomarker of PDAC.

**Supplementary Information:**

The online version contains supplementary material available at 10.1186/s13046-022-02310-8.

## Background

Pancreatic cancer (PC) is one of the most malignant cancers, and ranks as the fourth leading cause of cancer-related deaths, amounting to 4.5% of all cancer-related deaths globally [1]. Pancreatic ductal adenocarcinoma (PDAC) is responsible for over 80% of PC cases, with a 5-year survival of approximately 10% [2]. The poor prognosis of PDAC patients is associated with highly aggressive phenotype and early cancer recurrence and metastasis following surgical treatment. Surgical resection represents the only possibility of a cure for resectable and borderline resectable cases, while advancements in chemotherapy including the FOLFIRINOX regimen and gemcitabine plus nab-paclitaxel has improved the long-term outcomes of patients with metastasis [3]. Despite advances achieved in PDAC management in recent years, limited breakthroughs in the identification of effective biomarkers or treatment strategies have emerged. A deeper understanding of the molecular mechanism regulating cancer initiation and progression remains urgent to identify early diagnostic and prognostic biomarkers, as well as to discover novel therapeutic targets for PDAC.

Almost all PDAC patients carry at least one of four frequently-mutated driver genes including the oncogene KRAS and the tumor suppressors TP53, SMAD4, and CDKN2A [4, 5]. As a transcription factor, TP53 encodes the p53 protein that regulates ~ 300 target genes that coordinate diverse cellular effector functions including cell cycle arrest, apoptotic cell death, damaged DNA repair, free radical scavenging, and immune response regulation [6]. Importantly, p53 suppresses tumor initiation by transcriptionally inducing the critical cyclin-dependent kinase inhibitor p21 and essential pro-apoptotic modulators (PUMA, BAX, NOXA), leading to cell-cycle arrest and apoptosis of abnormal or damaged cells [7–9]. Mutation of TP53 or loss of p53 may induce tumor initiation and tumor growth [10]. Zinc finger matrin-type 1 (ZMAT1), maps to Xq22.1 and encodes a protein that contains three U1-like zinc fingers of Cys_2_His_2_(C2H2)-type zinc fingers family similar to those found in the nuclear matrix protein matrin 3. ZMAT1 belongs to a 5-member family (ZMAT1-5) in humans, in which all encoded proteins contain zinc finger domains, but are otherwise dissimilar [11]. Such associations have been reported between the zinc-finger proteins and cellular stress response pathways including those involved in DNA damage, cell cycle arrest, and apoptosis [12]. ZMAT3, a transcriptional target of p53, acts as a tumor suppressor by triggering cell cycle arrest and apoptosis [13]. However, the biological function of ZMAT1 in the context of tumorigenicity and tumor progression is unknown, as is its association with p53.

Herein, we found that ZMAT1 was down-regulated in PDAC and the reduced expression of ZMAT1 was associated with unfavorable clinicopathological characteristics and poor survival of PDAC. The loss of ZMAT1 promoted PDAC tumorigenicity and progression in vitro and in vivo. Moreover, we found ZMAT1 functioned in a p53-dependent manner and identified SIRT3 as an enhanced target and effector of ZMAT1 regulation of p53. The findings indicated a role for ZMAT1-SIRT3-p53 signaling during tumor growth, highlighting that ZMAT1 is a novel prognostic and therapeutic biomarker of PDAC.

## Methods

### Data acquisition

Gene expression data for 171 PDAC samples were obtained from The Cancer Genome Atlas (TCGA) database up to June 2021 (https://portal.gdc.cancer.gov/repository). The GSE62165 dataset based on the GPL13667 platform (contains 13 pancreas and 118 PDAC samples), the GSE62452 dataset based on the GPL6244 platform (contains 61 pancreas and 69 PDAC samples), the GSE15471 dataset based on the GPL570 platform (contains 36 pancreas and 36 PDAC samples), and the GSE16515 dataset based on the GPL570 platform (includes 16 pancreas and 36 PDAC samples) were downloaded from the Gene Expression Omnibus (GEO) database for expression validation (https://www.ncbi.nlm.nih.gov/geo/). Gene Expression Profiling Interactive Analysis (GEPIA, http://gepia.cancer-pku.cn/index.html) and Oncomine (https://www.oncomine.org) databases were also used to validate the transcriptional levels of ZMAT1 in PDAC and other cancer types [14, 15]. Correlation analysis of gene expression was performed using the GEPIA database using TCGA and Genotype-Tissue Expression Project (GTEx) data.

### Tissue samples and patient follow-up

For validation studies, tumor and adjacent normal tissue samples were obtained from 122 PDAC patients from the Guangdong Provincial People’s Hospital and the First Affiliated Hospital of Sun Yat-sen University (Validation cohort). All patients were followed to June 2021 and the median follow-up time was 12.3 months (range 3–42.3). Overall survival (OS) was defined as the date from surgical resection to death, while disease-free survival (DFS) was defined as the date from surgical resection to tumor metastasis or recurrence. The clinicopathological data of the enrolled patients, including sex, age, carbohydrate antigen 19–9 (CA19-9), lymph node metastasis, perineural invasion, TNM stage, and differentiation were manually collected. The study was approved by the Ethics Association of Guangdong Provincial People’s Hospital and the First Affiliated Hospital of Sun Yat-sen University. All enrolled patients provided written informed consent before participation. Each tissue sample was evaluated and diagnosed as PDAC by two different pathologists.

### Cox regression and Kaplan–Meier analysis

Univariate and multivariate Cox regression analyses were used to identify independent prognostic factors for PDAC prognosis in the validation cohort. Kaplan–Meier analyses and log-rank tests were conducted for the high- and low-ZMAT1 expression groups in both TCGA and validation cohorts to assess the ability to predict patient survival.

### Functional and pathway enrichment analysis

Genes co-expressed with ZMAT1 (|Spearman’s correlation coefficient|> 0.5 and *P* < 0.05) were screened from the cBioPortal database (https://www.cbioportal.org/). Co-expressed genes and ZMAT1-binding genes identified by Chromatin immunoprecipitation (ChIP) sequencing were integrated into DAVID 6.8 separately for Gene Ontology (GO) and Kyoto Encyclopedia of Genes and Genomes (KEGG) pathway analyses (https://david-d.ncifcrf.gov/). GO and KEGG analyses results were visualized using the R “ggplot2” package. Gene set enrichment analysis (GSEA) determined the gene sets and functional pathways that differed significantly between the high- and low-ZMAT1 expression groups. ZMAT1 expression was used as a phenotype label and 1000 gene set permutations were performed per analysis. The STRING database was used to perform functional enrichment analysis and to construct a protein–protein interaction (PPI) network, to subsequently identify P53-associated genes (https://string-db.org/cgi/) [16].

### Materials

Antibodies against p53 (ab26, 1/1000), p21 (ab109520, 1/1000), CDK2 (ab32147, 1/2000), CCNA2 (ab185619, 1/1000), BAD (ab32445, 1/1000), BAX (ab32503, 1/5000), Bcl-2 (ab32124, 1/1000), SIRT3 (ab217319, 1/1000), Ki-67 (ab16667, 1/200), Flag (ab205606, 1/30), Caspase-3 (ab32351, 1/5000), GAPDH (ab8245, 1/2000) and β-actin (ab8226, 1/5000) were from Abcam (Cambridge, UK). Anti-ZMAT1 (NBP1-81375, 1/100) was from Novus Biologicals (Colorado, USA) and anti-ZMAT1 (bs-4387R, 1/200) was from Bioss (Beijing, China). Tunel apoptosis assay kit (#8109) was from Cell Signaling Technology (Danfoss, USA). Pifithrin-α (PFT-α, S2929) was from Selleck (Texas, USA).

### Cell culture and transfection

HPDE6, PANC-1, BxPC-3, Capan-2, SW1990, and AsPC-1 cells lines were obtained from Procell (Wuhan, China). Cells were cultured in RPMI 1640 medium (Gibco, New York, USA), supplemented with 10% fetal bovine serum (FBS) at 37℃ with 5% CO2. PFT-α treatment was administered at a final concentration of 100 ng/ml for 24 h.

For the generation of cell lines with ZMAT1 overexpression or knockdown, the EGFP-tagged/3 × Flag hZMAT1-CMV Puro vector and three siRNAs targeting ZMAT1 were transfected into cells to overexpress and silence ZMAT1, respectively. Cell transfection was performed as previously described [17]. After antibiotic selection, the over-expression and depletion efficiency were assessed by Real-time quantitative polymerase chain reaction (RT-qPCR) and western blot. Reconstituted cells with stable overexpression of ZMAT1 and cells with depleted ZMAT1 were utilized for cell proliferation assays, cell migration assays, RT-qPCR, immunoblotting, and animal experiments as indicated below. The siRNA and PCR primer oligonucleotide sequences used in our study are shown in Tables S1-S2.

### Cell proliferation assays

Cell counting Kit-8 (CCK-8) and colony formation assays were used to determine cell viability. For CCK-8 assays, 1500 cells were seeded per well of a 96-well plate. At a specific timepoint, the CCK-8 solution was added to each well. The cells were then cultured at 37 °C under 5% CO2 and the absorbance (OD450) was assessed in a microplate reader after 0, 24, 48, and 72 h. For colony formation assays, cells of each cell type (2000 cells/ well) were seeded into 6-well plates, gently shaken, and cultured at 37 °C with 5% CO2 for 7–14 days. The medium was subsequently removed and the cells were stained with 0.1% crystal violet to quantify positive colonies (diameter > 30 µm).

### Cell migration assays

Transwell plates (Corning Costar, USA) were used for cell migration analysis. A total of 5 × 10^4^ cells were seeded in the upper chambers of Transwell plates in 200 µL of serum-free DMEM while DMEM containing 10% FBS was added to the lower chambers. After incubating for 24 h, migrated cells in the lower chambers were fixed in methanol and then stained with crystal violet. Migrated cells were imaged using an inverted microscope and quantified from three different fields.

Cell migration was also evaluated by the wound-healing assay. Cells (1 × 10^6^) were seeded into each well of a 6-well plate until 80–90% confluence. A sterile 200-µL pipette tip was then used to draw a wound in the cell monolayer, following which, the cells were washed twice with phosphate buffer saline (PBS). Images of the wounds were obtained at 0 and 48 h using a photomicroscope and wound closure was evaluated in at least three different fields using Image J 1.52 (National Institute of Health, USA).

### Flow cytometry of cell cycle and apoptosis

Flow cytometry was performed as previously described [17].

### Immunohistochemistry and immunofluorescence analysis

Paraffin-embedded sample tissues were consecutively cut into 4-μm slices and then mounted on glass slides for immunohistochemistry (IHC) staining. The sections were processed and stained using ZMAT1, p53, SIRT3, Ki67, and Tunel antibodies, respectively. After drying, the sections were examined and photographed under an Olympus BX63 microscope. ZMAT1 immunoreactivity was determined by staining intensity and distribution to obtain an H-score: < 50% staining was considered low expression, while ≧50% staining was considered high expression. The specimens were assessed independently by two pathologists. For immunofluorescence (IF) analysis, SW1990 cells were incubated with the primary antibody against ZMAT1 overnight at 4℃, followed by incubation with the secondary antibody for 2 h at 37℃ in the dark. DAPI was added for 10 min and images were taken with confocal microscopy (Olympus, FV3000) in a dark room.

### Real-time quantitative polymerase chain reaction and western blotting

Real-time quantitative polymerase chain reaction (RT-qPCR) was used to assess mRNA expression. Total RNA was extracted with TRIzol reagent (Life Technologies, USA) and then reversed transcribed to cDNA with a reverse transcription kit (Toyobo, Japan). RT-qPCR was performed with SYBR green mix (Toyobo, Japan) on the Light Cycler 480II (Roche, USA). The relative mRNA expression level was determined using the 2^−∆∆Ct^ method.

For western blot, cells were collected and lysed in an ice bath by RIPA buffer containing 1% phenylmethanesulfonylfluoride fluoride for 30 min to extract total protein. Protein concentrations were determined using the BCA protein assay. Equal amounts of protein were separated on an 8% SDS-PAGE gel and transferred onto a polyvinylidene fluoride membrane. After blocking for 1 h with a 3% BSA in TBST buffer, the membranes were probed with specific antibodies at 4℃ overnight. The membranes were washed by TBST buffer 5 times and incubated with secondary antibody marked with HRP-conjugated goat anti-rabbit IgG for 1 h at 25℃. Finally, the membranes were subjected to an enhanced chemiluminescence system (Pierce, USA) and the targeted protein bands were visualized.

### RNA sequencing

RNA was collected from SW1990/Vector and SW1990/ZMAT1-OV cells. After RNA reverse transcription, amplification, and quality control, the clustering of the index-coded samples was performed on a cBot Cluster Generation System using the TruSeq PE Cluster Kit v3-cBot-HS (Illumina, California, USA) according to the manufacturer’s instructions. After cluster generation, the library preparations were sequenced on an Illumina Novaseq platform (Sinotech Genomics, Shanghai, China) and analyzed with Counts v1.5.0-p3 and Stringtie v1.3.3b software. Differential mRNA expression analysis was carried out using the R package “DESeq2.” Differentially expressed genes (DEGs) were obtained as a Log_2_|Fold Change|> 1.5 and a false discovery rate < 5%. The RNA sequencing data were deposited to Sequence Read Archive (SRA) of National Center for Biotechnology Information (NCBI) and the accession numbers are SRR17968055, SRR17968056, SRR17968057, SRR17968058, SRR17968059 and SRR17968060.

### Luciferase reporter assay

The plasmid encoding the luciferase reporter gene for p53 (pGL3-p53) was purchased from Novel Biotechnology (Guangzhou, China). The reporter plasmid was transfected into HER-293 T cells together with blank plasmid or 0.01 μg, 0.05 μg, 0.1 μg, 0.5 μg, and 1 μg of ZMAT1. After 48 h of incubation, the luciferase activity was analyzed by Dual-Luciferase® Reporter (DLRTM) Assay System (Promega, Cat. No. E1910) and the relative luciferase activity was calculated by normalizing the firefly luciferase activity against the renilla luciferase activity. pGL3 vectors containing SIRT3 wild type and mutant promoters were chemically synthesized by Hedgehog BioScience and Technology Ltd (Shanghai, China). DNA segments were respectively cloned into the pGL3-basic vector purchased from Promega (Wisconsin, USA). Subsequently the transfection and luciferase reporter assay were performed as indicated as above.

### Chromatin immunoprecipitation (ChIP) assay, ChIP-based quantitative polymerase chain reaction, and ChIP-based sequencing

Chromatin immunoprecipitation (ChIP) assays were conducted using the Simple CHIP Plus Enzymatic Chromatin IP kit (Cell Signaling Technology, USA). SW1990/Vector and SW1990/ZMAT1-OV cells were cross-linked in 1% formaldehyde for 10 min and then glycine solution and ice-cold PBS were added to inactivate the formaldehyde and wash the cells, respectively. After centrifugation, the cells were lysed in 100 μg Lysis Buffer 1 to remove the cell membranes and then 0.25 μL Micrococcal Nuclease was added for 15 min. Finally, 10 µL of MNase Stop Solution was added to stop the reaction and the samples were centrifuged to recover the nuclei. Next, the samples were lysed in 50 µL of Lysis Buffer 2 on ice for 15 min and centrifuged to obtain the supernatant containing the digested chromatin. Aliquots of 5 µL of the supernatant were stored at -20 °C and used as the input sample. The remaining 45 µL of supernatant was supplemented with 450 µL of 1 × IP Dilution Buffer, and subsequently incubated with 15 µL of anti-Flag antibody or 1 µL of Normal Rabbit IgG at 4 °C, overnight, respectively. After incubation with 20 µL of ChIP Grade Protein A/G Plus Agarose for 1 h on a rocking platform, each sample was washed with IP Wash buffer 1, IP Wash buffer 2, and IP Wash buffer 3. The samples were incubated with 150 µL of 1 × IP Elution Buffer at 65 °C for 30 min, and then placed in a collection tube with 2 µL of 20 mg/ml Proteinase K and 6 µL of 5 M NaCl. Meanwhile the input sample was thawed and added to 150 µL of the 1 × IP Elution buffer, 2 µL of 20 mg/ml Proteinase K, and 6 µL of 5 M NaCl. Input and IP samples were incubated in a 65 °C heat block for 1.5 h to reverse cross-links. The ChIP DNA and input DNA were purified using a DNA Clean-Up Column, rinsed with the DNA Column Wash Buffer, and eluted using the DNA Column Elution Solution.

The purified DNA was used for subsequent RT-qPCR and sequencing. RT-qPCR was performed with the SYBR Green Mix (Toyobo, Japan) on a Light Cycler 480II (Roche, USA). Sequences were generated using the Illumina NovaSeq 6000 genome analyzer and aligned to the Human genome (HG19) using BOWTIE software (V2.2.7). The mapped reads were used for peak detection by Model-based Analysis of ChIP-Seq (MACS) v1.4.2 software. Statistically significant ChIP-enriched regions (peaks) were identified by comparison of IP vs Input or comparison to a Poisson background model (cut-off *P* = 1 × 10^–3^).

### Animal experiments

A total 1 × 10^6^ SW1990/ZMAT1-OV cells and control cells were subcutaneously injected to BALB/c nude mice to establish the xenograft mouse models (*n* = 6 per group). The tumor sizes and volumes of mice were recorded by a digital caliper every 10 days. On Day 60, the mice were sacrificed and the tumors were dissected, photographed, weighed, and subjected to IHC analysis with the indicated antibodies. All 6-week old male BALB/c nude mice were purchased from GemPharmatech (Jiangsu, China). The assignment of mice to different groups was done randomly. All animal experiments were approved by the Ethics Association of Guangdong Provincial People’s Hospital.

### Statistical analysis

The statistical analysis of continuous parameters between the two groups was determined by Student’s t-test, while one-way and repeated-measures analysis of variance (ANOVA) was used to compare multiple groups. χ^2^ test or Fisher’s exact test was used to explore qualitative variables as appropriate. Spearman’s correlation was performed to analyze the correlation between variables. All statistical analyses were performed using R software version 4.0.1 (https://www.r-project.org/) and SPSS version 24.0 (SPSS, Inc., Chicago, IL, USA). A *P* < 0.05 was considered statistically significant unless otherwise specified.

## Results

### ZMAT1 was down-regulated in PDAC

**Fig. 1:**
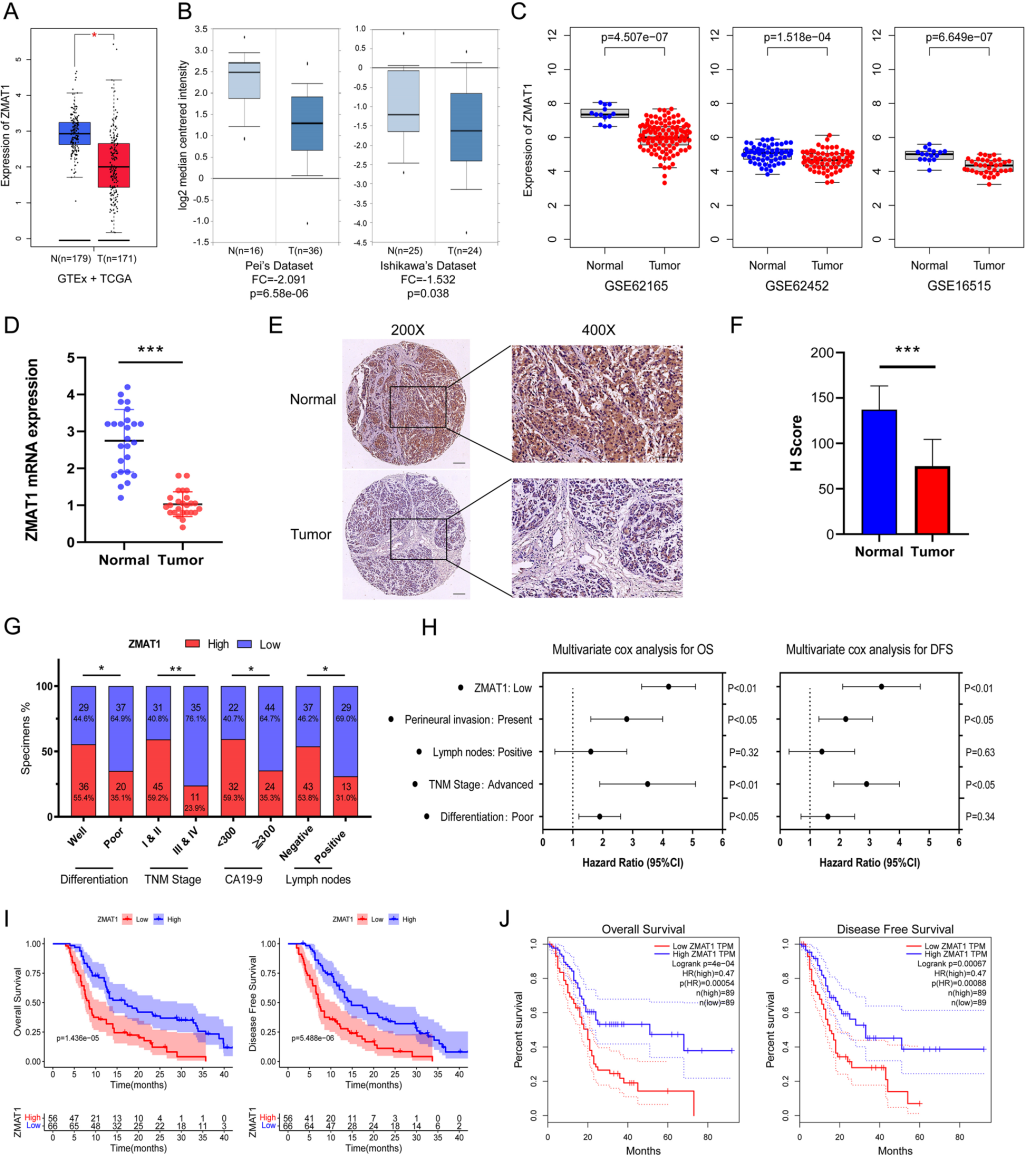
ZMAT1 is down-regulated and correlates with unfavorable clinical characteristics and adverse outcome in Pancreatic Ductal Adenocarcinoma (PDAC). **A** Down-regulation of ZMAT1 in PDAC was identified in GEPIA database. **B** Down-regulation of ZMAT1 was identified in PDAC in Oncomine database (Pei’s dataset and Ishikawa’s dataset). **C** Down-regulation of ZMAT1 was identified in PDAC in three individual GEO datasets (GSE62165, GSE62452 and GSE16515). **D** The mRNA low-expression levels of ZMAT1 were identified in PDAC tissues and normal pancreas tissues of 25 samples. **E** Representative images of ZMAT1 staining in PDAC specimens and normal pancreas tissues. **F** Immunohistochemistry staining showed the protein levels of ZMAT1 were down-regulated in PDAC tissues. **G** ZMAT1 expression of PDAC was significantly correlated with differentiation, TNM stage, CA19-9 index and lymph nodes metastasis. **H** Multivariate Cox regression analyses showed low expression of ZMAT1 was independent risk factor for overall survival (OS) and disease-free survival (DFS) of 122 PDAC patients from validation cohort. **I**-**J** Kaplan–Meier analyses showed PDAC patients with high expression of ZMAT1 had superior OS and DFS than those with low expression in both TCGA cohort (**I**) and validation cohort (**J**). T, tumor; N, normal; OS, overall survival; DFS, disease-free survival. CA19-9, carbohydrate antigen 19–9. All * *P*-value < 0.05, ** *P*-value < 0.01, *** *P*-value < 0.001. Scale bars: 200 μm. *P*-values were determined by Non-parametric Mann–Whitney U-test in A-C. *P*-values were assessed by two-tailed t-tests in D and F. *P*-values were determined by χ2 tests or Fisher’s exact tests in G. The Hazard Ratios (HR) and *P*-values by the log-rank (Mantel-Cox) test are calculated in H-J

To evaluate the role of ZMAT1 in cancer progression, bio-informatics analysis was performed to explore the expression of ZMAT1 across different cancer types. As revealed by GEPIA tool in Fig. 1A and S1A, ZMAT1 expression was down-regulated in most cancer types compared to adjacent normal tissues especially in PDAC. Pei’s dataset (*P* = 6.58e-06) and Ishikawa’s dataset (*P* = 0.038) from the Oncomine Platform revealed that ZMAT1 expression was decreased in PDAC tumors compared to normal pancreas samples (Fig. 1B). Low expression of ZMAT1 in PDAC were also identified in three individual GEO datasets including GSE62165 (*P* = 4.507e-07), GSE62452 (*P* = 1.518e-04), and GSE16515 (*P* = 6.649e-07) (Fig. 1C). In addition, RT-qPCR analysis was performed on 25 fresh PDAC tissues and normal pancreas tissues, and the results validated the down-regulation of mRNA levels of ZMAT1 in tumor tissues (Fig. 1D). As expected, the H scores of IHC on 122 pairs of PDAC and pancreas tissues from the validation cohort also confirmed that the protein levels of ZMAT1 were decreased in PDAC tissues (Fig. 1E-F).

### Reduced ZMAT1 expression correlated with unfavorable clinical characteristics and adverse outcome in PDAC

To assess the clinical value of ZMAT1, 122 PDAC patients from the validation cohorts were divided into high- and low-ZMAT1 groups according to the results of IHC analysis. The correlations between the expression of ZMAT1 and the clinicopathological characteristics of PDAC are shown in Fig. 1G and Table S3. Low ZMAT1 expression of PDAC was significantly correlated with poor tumor differentiation (*P* < 0.05), advanced TNM stage (*P* < 0.01), high CA19-9 index (*P* < 0.05), and positive lymph nodes metastasis (*P* < 0.05). Next, univariate and multivariate Cox regression analyses were performed in the validation cohort, which revealed that low ZMAT1 expression was an independent risk factor for OS and DFS of PDAC patients (Fig. 1H and Table S4 and S5). Kaplan–Meier analyses results indicated that PDAC patients with low ZMAT1 expression had inferior OS and DFS than those with high expression in both the TCGA (*P* < 0.001) and validation cohorts (*P* < 0.001) (Fig. 1 I-J). Further, patients with low ZMAT1 expression also had poor prognosis in other cancer types including adrenocortical carcinoma, head and neck squamous cell carcinoma, lung adenocarcinoma, mesothelioma, and skin cutaneous melanoma (Fig. S1B). Taken together, these findings indicated that ZMAT1 was an effective prognostic indicator for PDAC.

### ZMAT1 inhibited proliferation and migration of PDAC cells

**Fig. 2:**
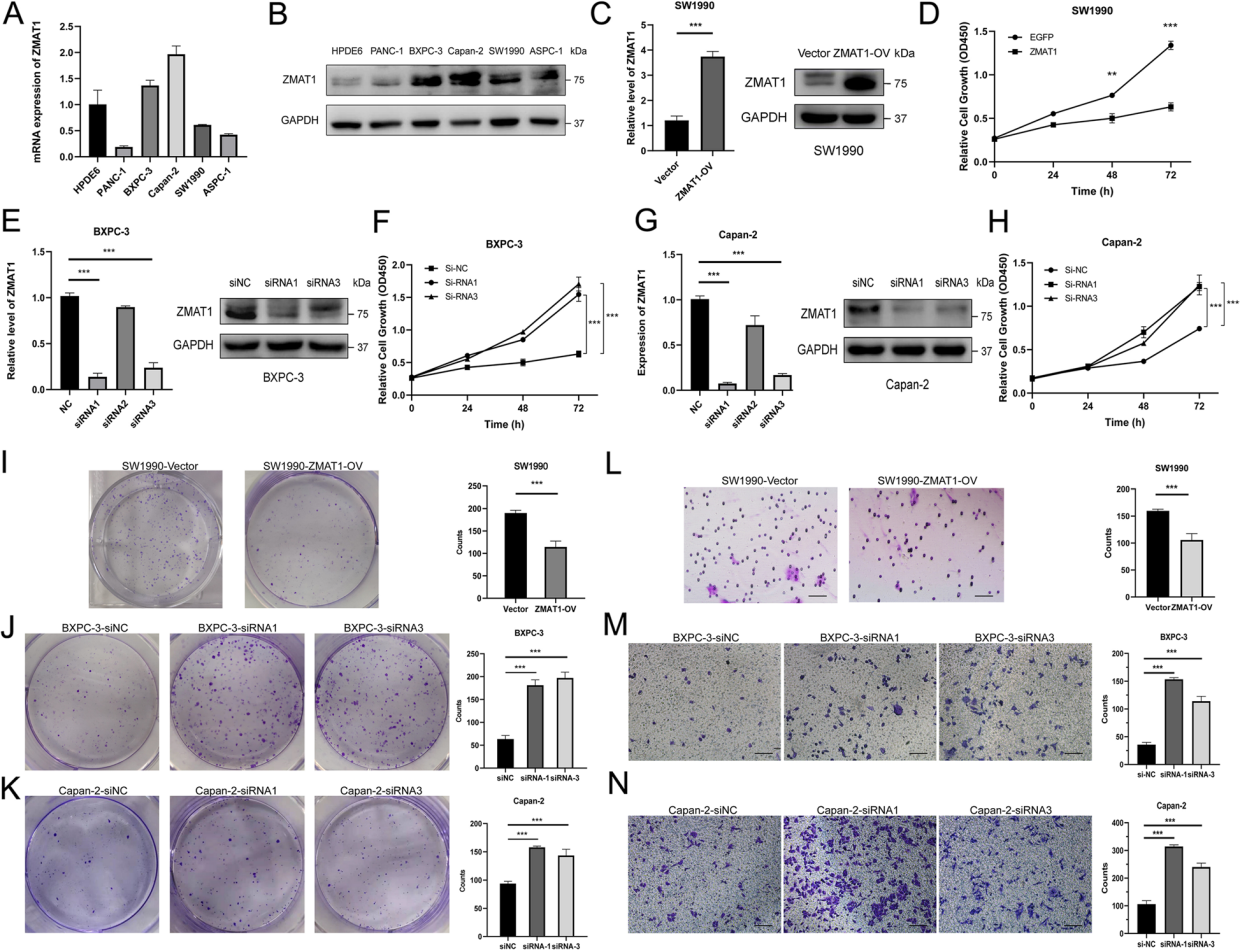
ZMAT1 inhibits proliferation and migration of Pancreatic Ductal Adenocarcinoma (PDAC) cells. **A**-**B** Real-time quantitative polymerase chain reaction (RT-qPCR) (**A**) and western blot (**B**) showed ZMAT1 was highly expressed in BXPC-3 and Capan-2 cells, while relatively low-expressed in SW1990 cells among PDAC cell lines. **C** RT-qPCR and western blot were used to detect the over-expression of ZMAT1 in SW1990 cells. **D** CCK-8 assays showed that ZMAT1 over-expression reduced the proliferation in SW1990 cells. **E** RT-qPCR and western blot were used to detect the down-regulation of ZMAT1 in BXPC-3 cells transfected with ZMAT1-siRNA. **F** CCK-8 assays showed that ZMAT1 down-regulation promoted the proliferation in BXPC-3 cells. **G** RT-qPCR and western blot were used to detect the down-regulation of ZMAT1 in Capan-2 cells transfected with ZMAT1-siRNA. **H** CCK-8 assays showed that ZMAT1 down-regulation promoted the proliferation in Capan-2 cells. **I**-**K** Colony formation assays showed that ZMAT1 over-expression reduced the proliferation in SW1990 cells (**I**), while ZMAT1 knockdown promoted the proliferation in BXPC-3 (**J**) and Capan-2 cells (**K**). **L**-**N** Transwell assays showed that ZMAT1 over-expression reduced the migration in SW1990 cells (**L**), while ZMAT1 knockdown promoted the migration in BXPC-3 (M) and Capan-2 cells (**N**). All * *P*-value < 0.05, ** *P*-value < 0.01, *** *P*-value < 0.001. Scale bars: 100 μm. *P*-values were assessed using two-tailed t-tests and ANOVA followed by Dunnett’s tests for multiple comparison in C-N. All figures represent mean ± SD from three independent experiments

The mRNA and protein expression levels of ZMAT1 in several PDAC cell lines were detected. ZMAT1 was relatively highly expressed in BXPC-3 and Capan-2 cell lines, but had lower expression in SW1990 cells (Fig. 2A-B). To better understand the biological roles of ZMAT1 in PDAC, we established a SW1990 cell line with stable ZMAT1 over-expression (OV) (SW1990/ZMAT1-OV) to analyze gain-of-function activity of ZMAT1 (Fig. 2C). Three si-ZMAT1 were transfected into BXPC-3 and Capan-2 cells to obtain BXPC-3/ZMAT1-KD and Capan-2/ZMAT1-KD cell lines for loss-of-function analysis via gene knockdown (KD) (Fig. 2E and 2G). The morphology of SW1990, BXPC-3 and Capan-2 after over-expression or down-regulation was showed in Fig. S2A-C. Following RT-qPCR and western blot analyses that validated the expression levels of ZMAT1, CCK-8, and colony formation assays were conducted on these three cell lines to assess changes in cell proliferation. As indicated in Fig. 2D and 2I, up-regulation of ZMAT1 significantly inhibited SW1990 cell proliferation. In contrast, down-regulation of ZMAT1 enhanced the proliferative ability of BXPC-3/ZMAT1-KD and Capan-2/ZMAT1-KD cell lines (Fig. 2F, 2H, and 2J-K). Next, Transwell assays and wound healing assays were carried out to evaluate the functions of ZMAT1 on cell migration in vitro. The Transwell assays showed the migration of tumor cells was repressed in the ZMAT1 over-expressed cell line, while silencing of ZMAT1 augmented cellular migration in knock-down cell lines (Fig. 2M-N). Further, we observed that ZMAT1 over-expression suppressed cell motility in wound-healing assays and decreased cell scattering at the wound edge (Fig. S3A), whereas knock-down of ZMAT1 promoted wound closure (Fig. S3B-C). These results indicated that loss of ZMAT1 promoted proliferation and migration of PDAC cells in vitro.

### ZMAT1 regulates cell cycle progression and cell apoptosis in PDAC

**Fig. 3:**
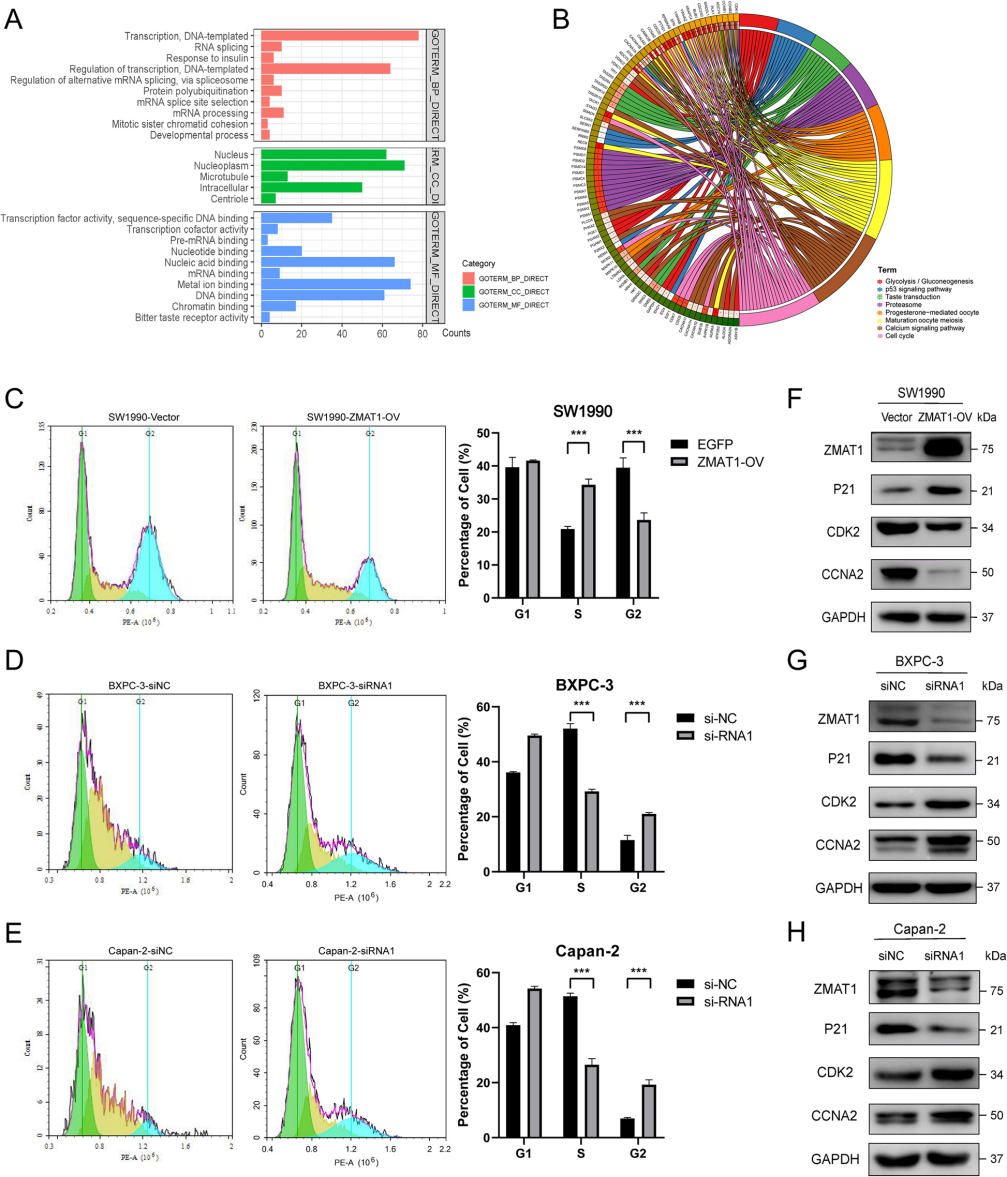
Over-expression of ZMAT1 arrests the cell cycle in Pancreatic Ductal Adenocarcinoma (PDAC) cells. **A** Gene Ontology (GO) enrichment analysis of ZMAT1 based on TCGA cohort. **B** Kyoto Encyclopedia of Genes and Genomes (KEGG) pathway analysis of ZMAT1 based on TCGA cohort. **C**-**E** Flow cytometry showed over-expression of ZMAT1 triggered G2/S blockade in SW1990 cells (**C**), while depletion of ZMAT1 increased the percentage of S phase in BXPC-3 (**D**) and Capan-2 cells (**E**). **F**–**H** Western blot showed over-expression of ZMAT1 up-regulated p21 and decreased CDK2 and CCNA2 in SW1990 cells (**F**), whereas ZMAT1 knockdown declined p21 and increased CDK2 and CCNA2 in BXPC-3 (**G**) and Capan-2 cells (**H**). All * *P*-value < 0.05, ** *P*-value < 0.01, *** *P*-value < 0.001. *P*-values were assessed using two-tailed t-tests in C-E. All figures represent mean ± SD from three independent experiments

To further investigate ZMAT1 function, 484 co-expressed genes were identified using the cBioPortal database and were submitted into DAVID 6.8 for GO and KEGG functional analyses. In the GO analysis, the GO terms and enriched pathways showed that ZMAT1 was mainly enriched in the nucleus and RNA splicing pathways and may regulate, together with its co-expressed genes, cell cycle-related processes, including nucleic acid binding and transcription (Fig. 3A), while its role in KEGG analysis might be involved in “Glycolysis/Gluconeogenesis,” “p53 signaling,” “Taste transduction,” “Proteasome activity,” “Progesterone-mediated oocyte maturation,” “Oocyte meiosis,” “Calcium signaling pathway,” and “Cell cycle regulation” (Fig. 3B).

Based on the GO and KEGG analyses, flow cytometry was performed to determine the specific effects of ZMAT1 on cell cycle and apoptosis in PDAC. The results showed SW1990/ZMAT1-OV cells had more population of S phase and less of G2 phase (Fig. 3C), while the silencing of ZMAT1 resulted in less population of S phase and more of G2 phase in BXPC-3/ZMAT1-KD and Capan-2/ZMAT1-KD cell lines (Fig. 3D-E). ZMAT1 was closely associated with cyclin dependent kinases (CDKs) and cyclins in TCGA dataset (Fig. S3A). Indeed, we found that the protein level of p21 was elevated, whereas the levels of CDK2 and CCNA2 were decreased in SW1990/ZMAT1-OV cells compared with the control group (Fig. 3F). In contrast, the down-regulated expression of p21 and over-expressions of CDK2 and CCNA2 were detected in knock-down cell lines (Fig. 3G-H). Since CDK2 and CCNA2 regulated the completion of S phase [18] and they were decreased in ZMAT1-OV cells, the results in Fig. 3 indicated that ZMAT1 could impair S/G2 progression in PC cell cycle by regulating p21 expression.

**Fig. 4:**
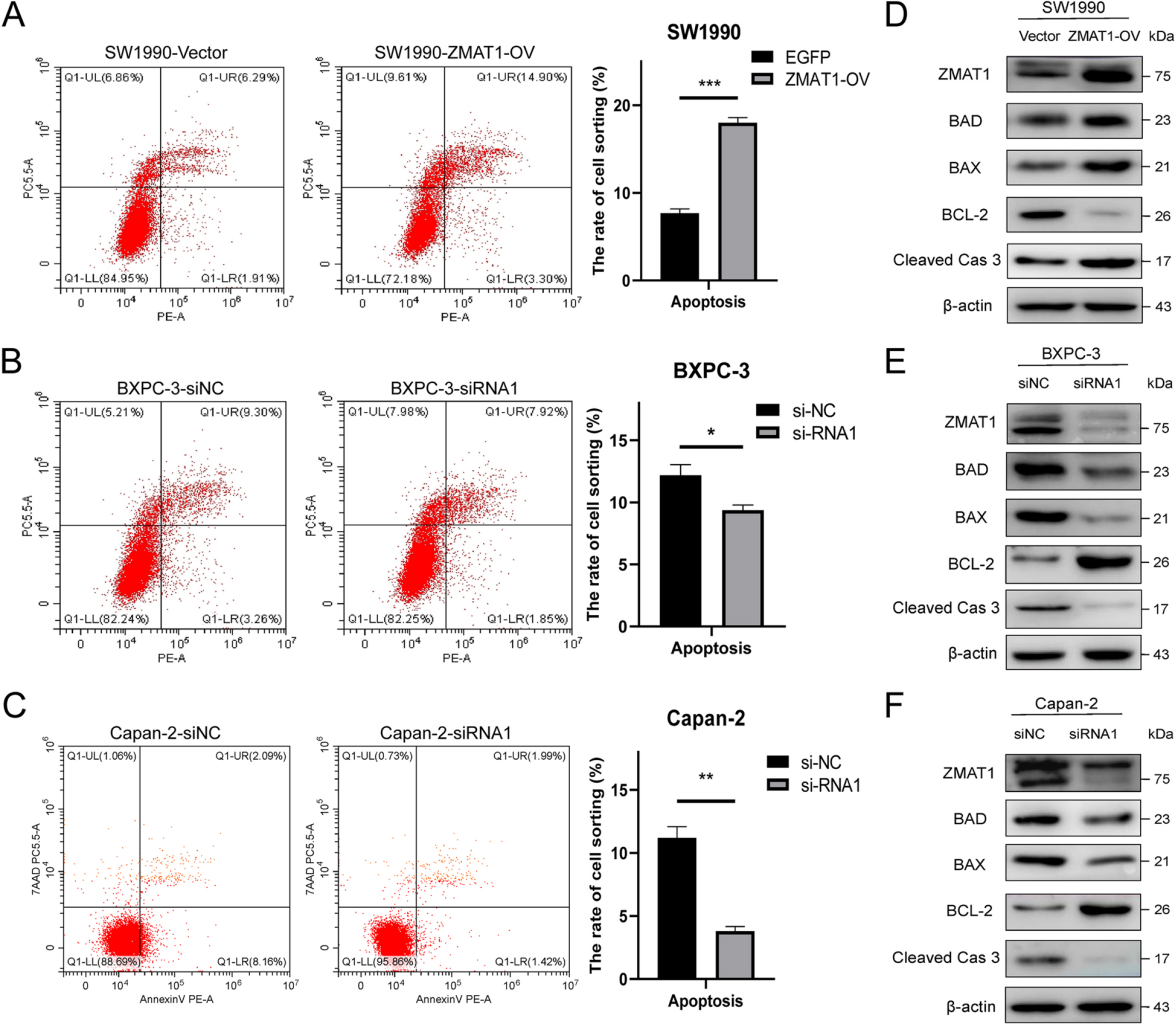
Over-expression of ZMAT1 promotes apoptosis in Pancreatic Ductal Adenocarcinoma (PDAC) cells. **A** Flow cytometry showed that over-expression of ZMAT1 increased the percentage of apoptotic SW1990 cells. **B**-**C** Flow cytometry showed that ZMAT1 deletion reduced the percentage of apoptotic SW1990 cells. **D**-**F** Western blot showed over-expression of ZMAT1 up-regulated BAD, BAX, Cleaved Caspase 3 and declined Bcl-2 in SW1990 cells (**D**), whereas ZMAT1 knockdown reduced BAD, BAX, Cleaved Caspase 3 and increased Bcl-2 in BXPC-3 (**F**) and Capan-2 cells (**G**). All * *P*-value < 0.05, ** *P*-value < 0.01, *** *P*-value < 0.001. P-values were assessed using two-tailed t-tests in C-E. All figures represent mean ± SD from three independent experiments

As shown in Fig. 4A-C, flow cytometry also suggested that the up-regulation of ZMAT1 increased the percentage of apoptotic SW1990 cells, while ZMAT1 depletion reduced the proportion of apoptotic BXPC-3 and Capan-2 cells. Considering the correlations between ZMAT1 and apoptosis modulators revealed by TCGA data (Fig. S3A), we performed western blot to confirm that the over-expression of ZMAT1 significantly upregulated BAD, BAX and Cleaved Caspase 3 levels and inhibited Bcl-2 expression in SW1990 cells (Fig. 4D). Conversely, the knock-down of ZMAT1 decreased BAD, BAX and Cleaved Caspase 3 expression and increased Bcl-2 expression in BXPC-3 and Capan-2 cells (Fig. 4E-F). These results demonstrated that ZMAT1 activated the apoptosis pathway in PC cells.

### ZMAT1 functioned in a p53-dependent manner

**Fig. 5:**
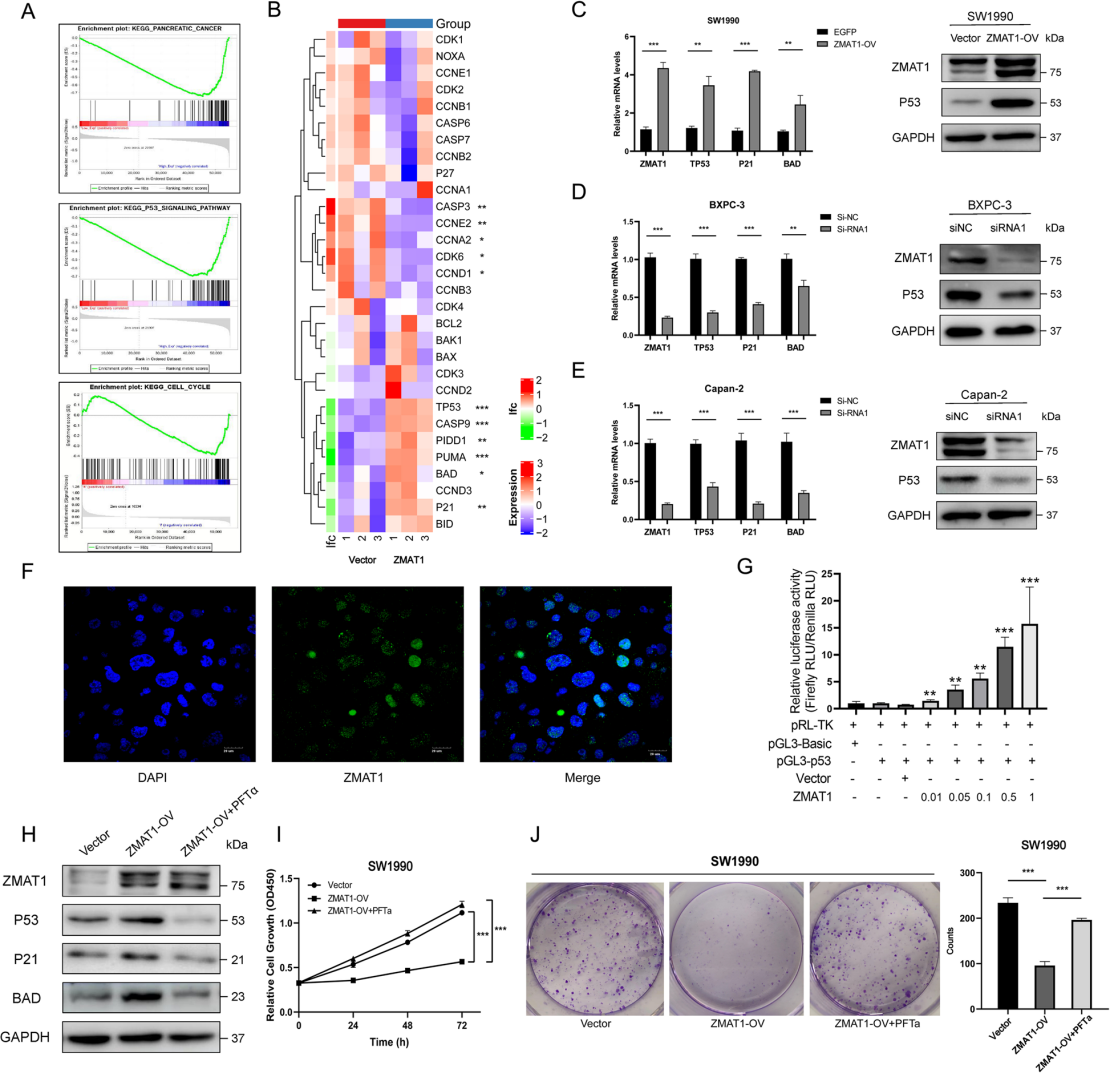
ZMAT1 functions in a p53-dependent manner. **A** Gene set enrichment analysis (GSEA) of RNA sequencing on SW1990/Vector and SW1990/ZMAT1-OV cells groups. **B** Heatmap of RNA sequencing showed differential expression levels of key nodes in p53-assicoated cell cycle and apoptosis pathways. **C**-**D** RT-qPCR and western blot were used to detect the p53 levels in ZMAT1 over-expressed (**C**) and knockdown cells (**D**). **E** Double-label immunofluorescence staining for the intracellular localization of ZMAT1 and p53 in SW1990 cells. **F** luciferase activity assays were performed on 293 T cells with co-transfection of pGL3-p53 with 0.01 μg, 0.05 μg, 0.1 μg, 0.5 μg and 1 μg of vector encoding ZMAT1. **G** SW1990/ZMAT1-OV cells were treated with Pifithrin-α for 24 h and the protein levels of ZMAT1, p53, p21, and BAD were analyzed by immunoblotting with the indicated antibodies. **H** CCK-8 assays were performed on SW1990/ZMAT1-OV cells after treating with Pifithrin-α for 24 h. **I** Colony formation assays were performed on SW1990/ZMAT1-OV cells after treating with Pifithrin-α for 24 h. All * *P*-value < 0.05, ** *P*-value < 0.01, *** *P*-value < 0.001. P-values were assessed using two-tailed t-tests and ANOVA followed by Dunnett’s tests for multiple comparison in B, C, D, E, G, J and K. All figures represent mean ± SD from three independent experiments

To investigate the downstream pathways or molecules of ZMAT1, RNA sequencing was performed by TruSeq PE Cluster Kit v3-cBot-HS (Illumina) on SW1990/Vector and SW1990/ZMAT1-OV cells groups. In total, 743 genes were identified as differentially expressed genes (DEGs) (|Log_2_FC|> 0.5) and the GSEA analysis was performed to identify gene sets and pathways distinguishing high- and low-ZMAT1 expression groups. As expected, significantly enriched biological processes included “Pancreatic Cancer,” “P53 Signaling Pathway,” and “Cell Cycle regulation” (Fig. 5A). We analyzed the expression of key molecules in the P53-associated cell cycle- and apoptosis-related pathways. Based on the sequencing data, the expressions of P53, P21, BAD, PIDD1, PUMA, and CASP9 were up-regulated, while CDK6, CCNA2, CCND1, CCNE2, and CASP3 were down-regulated in ZMAT1-OV cells (Fig. 5B). Next, we detected the expressions of p53 protein and its downstream effectors to further investigate the correlations between ZMAT1 and p53. The results of RT-qPCR and western blot suggested the mRNA and protein expression of p53 and its downstream effectors, p21 and BAD were enhanced with the over-expression of ZMAT1 (Figs. 3D, 4D, and 5C), whereas these declined after ZMAT1 deletion (Figs. 3F, 4F, 5D and 5E), indicating a transcriptional-level regulation of ZMAT1 on p53.

Since ZMAT1 is mainly localized in nucleus and contains the C2H2 zinc finger domain (Fig. 5F), we hypothesized ZMAT1 might bind to the promoter region of P53 and affect its transcription directly. To test this hypothesis, we performed ChIP sequencing and luciferase assays. We found ZMAT1 physically interacted with some DNA fragments but not those of P53. However, by transiently co-transfecting pGL3-p53 or pGL3-basic with a vector encoding ZMAT1 or empty vector into 293 T cells and conducting luciferase assays, the luciferase activity gradually increased in cells treated with pGL3-p53 and the increased concentration of ZMAT1 (Fig. 5G), indicating ZMAT1 induced the transcription of P53. Combining the results of ChIP sequencing and the luciferase assay, we concluded ZMAT1 influenced p53 expression at the transcriptional level, but in an indirect way.

We further explored whether ZMAT1 affected the malignant phenotype of PC cells in a p53-dependent manner. The p53 inhibitor Pifithrin-α (PFT-α) was used to inhibit p53 expression and activity, which was validated by several studies [19, 20]. We found that treating with PFT-α in ZMAT1-OV cells significantly diminished ZMAT1-induced expression of p53, p21, and BAD (Fig. 5H). CCK-8 assays showed that PFT-α could rescue ZMAT1-induced decrease of cell viability in SW1990 cells (Fig. 5I), while colony formation assays also demonstrated that treatment with PFT-α relieved the cell proliferation suppression induced by ZMAT overexpression (Fig. 5J). Taken together, these results showed that ZMAT1 regulated the malignant phenotype of PC cells in a p53-dependent manner.

### SIRT3 was a ZMAT1 downstream effector in PDAC

**Fig. 6:**
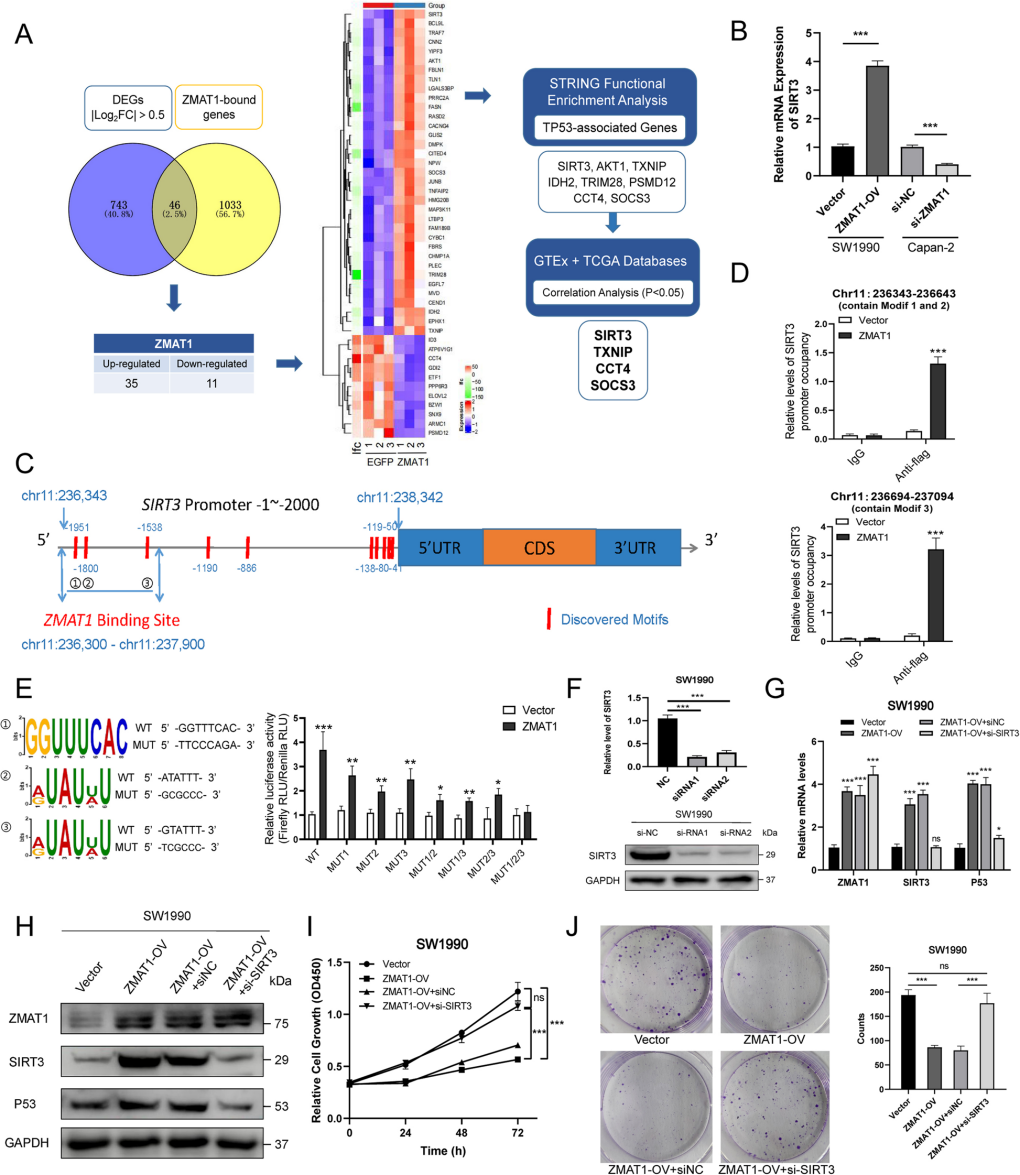
Identification of ZMAT1 downstream effectors in Pancreatic Ductal Adenocarcinoma (PDAC). **A** Flow diagram of the bioinformatic analyses and selection of downstream gene candidates targeted by ZMAT1. Venn diagram depicts the overlap between 789 differentially expressed genes (DEGs) upon ZMAT1 over-expression (blue) and 1079 ZMAT1-bound genes (yellow). Among 46 overlapping genes, 35 genes were up-regulated and 11 were down-regulated in SW1990/ZMAT1-OV cells. 4 downstream candidate genes were finally selected through STRING functional enrichment analyses and correlation analyses. **B** RT-qPCR analyses showed the relative mRNA levels of SIRT3 in ZMAT1 over-expression and deletion cell lines. **C** Schematic depicting the detailed information of ZMAT1-binding sites and discovered modifs (highlighted in red) on SIRT3 gene. **D** ChIP-qPCR analyses of ZMAT1 binding on SIRT3 promoter in SW1990/ZMAT1-OV cells. **E** Luciferase activity assays was performed in 293 T cells with transient expression of the WT or MUT SIRT3 gene promoter and ZMAT1. **F** RT-qPCR and western blot were used to detect the deletion of SIRT3 in SW1990 cells transfected with two SIRT3-siRNA. (**G**-**H**) SW1990/ZMAT1-OV cells were transfected with SIRT3-siRNA and the mRNA and protein levels of ZMAT1, SIRT3 and p53 were analyzed by RT-qPCR (**G**) immunoblotting (**H**). **I** CCK-8 assays were performed on SW1990/ZMAT1-OV cells transfected with SIRT3-siRNA. **K** Colony formation assays were performed on SW1990/ZMAT1-OV cells transfected with SIRT3-siRNA. DEGs, differentially expressed genes. All * *P*-value < 0.05, ** *P*-value < 0.01, *** *P*-value < 0.001. *P*-values were assessed using two-tailed t-tests and ANOVA followed by Dunnett’s tests for multiple comparison in B, D, E, F, G, I and J. All figures represent mean ± SD from three independent experiments

We next tried to identify the mechanism through which ZMAT1 regulates p53 expression. Although our findings showed that ZMAT1 did not bind to P53 directly, ChIP-sequencing identified 2567 significant ZMAT1-binding peaks and 1079 putative ZMAT1-binding genes. To assess the functions of ZMAT1-binding genes, GO functional analysis was performed and the top 10 biological process identified included “Regulation of signal transduction by p53 class mediator,” “Regulation of cell growth,” “Mitotic cell cycle checkpoint,” “Cell cycle,” and “Apoptotic process,” which highly coincided with our findings. We overlaid 1079 ZMAT1-binding genes identified by ChIP-seq with 743 DEGs obtained by RNA sequencing on SW1990/ZMAT1-OV cells using Venn diagram analysis, and the results showed that 46 genes were common to both groups (Fig. 6A). Next, these 46 genes were integrated to STRING to obtain TP53-associated genes. Based on the results of functional enrichment analyses, we identified eight TP53-associated genes (SIRT3, AKT1, TXNIP, IDH2, TRIM28, PSMD12, CCT4, and SOCS3) (Figs. 6Aand S4B). Next, we analyzed the correlation of expression of the selected 8 genes with ZMAT1 in TCGA dataset using the GEPIA tool and verified that SIRT3, TXNIP, CCT4, and SOCS3 showed significant correlations with ZMAT1 (Figs. 6A and S4C). Subsequently, the results of RT-qPCR showed SIRT3 had significantly higher and lower expression in the ZMAT1 over-expression and depletion groups, respectively, which was consistent with the RNA sequencing data (Fig. 6B). Thus, we defined SIRT3 as a P53-associated gene, which might be activated by ZMAT1 transcriptionally and was selected for further study.

SIRT3, a potential tumor suppressor in some solid cancers [21], was also down-regulated in PDAC (Fig. S5A-B). Notably, PDAC patients with high SIRT3 expression levels were associated with better OS and DFS (Fig. S5C). As shown in the ChIP-seq analysis, the SIRT3 gene contained a ZMAT1-binding region (chr11:236,300-chr11:237,900) which included three potential binding sites in its promoter region (Fig. 6C). Via ChIP-qPCR analysis we verified ZMAT1 recruitment to these two DNA fragments (chr11:236,343-chr11:237,643 and chr11:236,694-chr11:237,094) that contained three potential binding sites within the SIRT3 gene sequence (Fig. 6D), indicating binding of ZMAT1 to SIRT3 at specific sites. In addition, we cloned and mutated three potential mutated motifs to identify the binding site of ZMAT1 on the SIRT3 promoter region into pGL3 vectors respectively and carried out luciferase reporter assays in SW1990 cells (Fig. 6E). Consistent with this result, luciferase assays showed that significantly increased luciferase activity upon SIRT3 binding could be observed in cells transfected with any of the three segments (Fig. 6E). When the three binding sites were mutated simultaneously, ZMAT1 was unable to induce any activity within the SIRT3 promoter.

To determine whether ZMAT1 induced p53 expression was dependent on SIRT3 expression, siRNAs targeting SIRT3 were transfected into SW1990/ZMAT1-OV cells and RT-qPCR and western blot assays were used to identify SIRT3 depletion (Fig. 6F). As shown in Fig. 6G and H, the increased mRNA and protein of p53 in ZMAT1-OV cells were reversed by SIRT3 depletion. CCK-8 and colony formation assays also showed that SIRT3 depletion could rescue cells growth inhibition in ZMAT1-OV cells (Fig. 6I-J). Collectively, the results in Fig. 6 suggested that ZMAT1 promoted the transcription of SIRT3 by binding to three sites in its promoter, and subsequently up-regulated p53 and inhibited cancer cell proliferation.

### Overexpression of ZMAT1 suppressed tumor growth in vivo

**Fig. 7:**
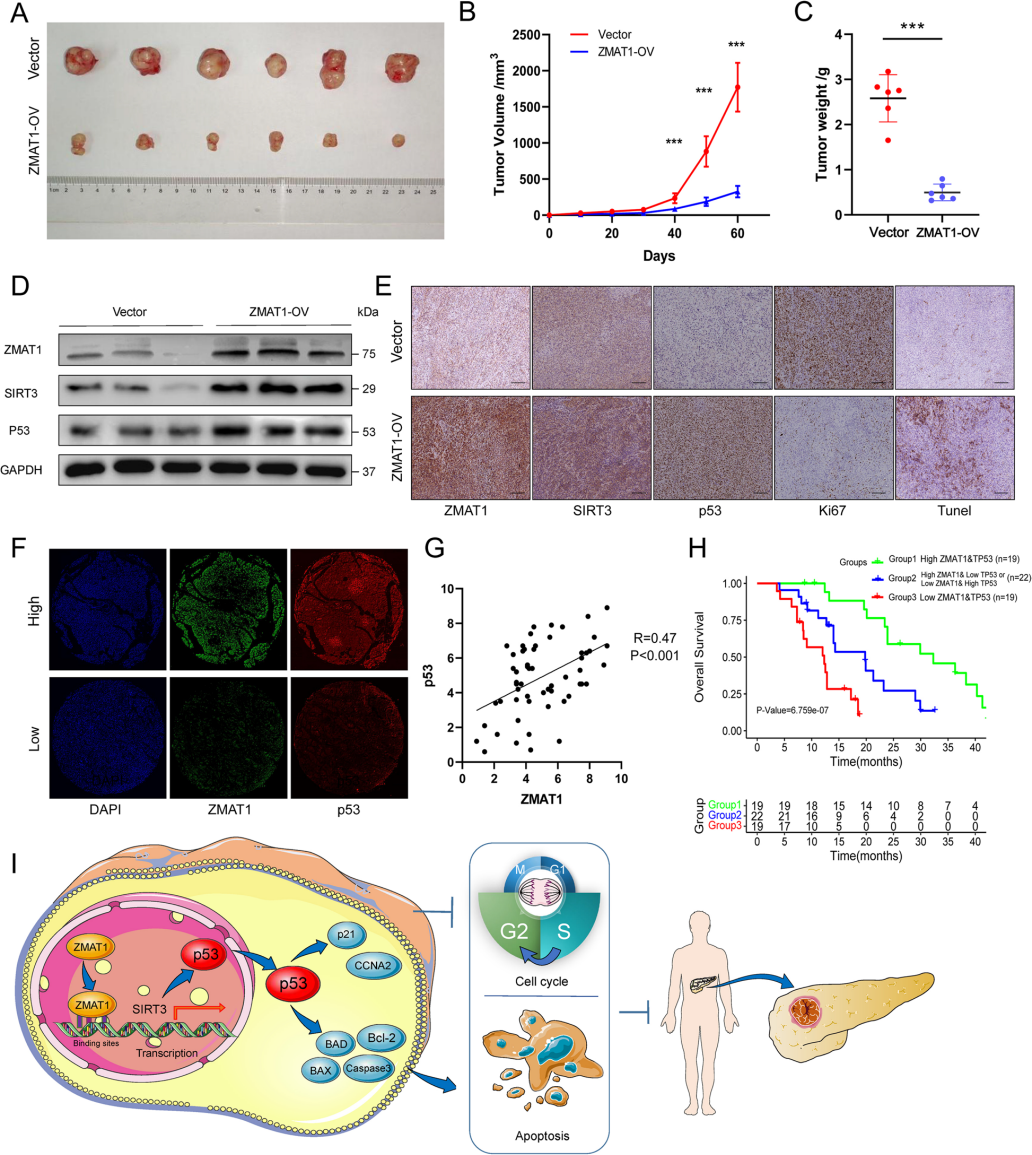
ZMAT1 correlates with p53 in Pancreatic Ductal Adenocarcinoma (PDAC). **A** BALB/c-nudes (*n* = 6 per group) were sacrificed 60 days after the injection and tumors dissected from respective groups were shown. **B** Tumor growth curves after the injection of SW1990/Vector cells and SW1990/ZMAT1-OV cells. Tumor volume was calculated every 10 days. **C** Tumor weight was measured in ZMAT1-OV and control groups. **D** The protein levels of ZMAT1, SIRT3 and p53 of tumors were analyzed by immunoblotting with the indicated antibodies. **E** IHC staining of ZMAT1, SIRT3, p53, Ki67 and Tunel in tumors from ZMAT1-OV and control groups. **F** Representative images of double-label immunofluorescence (IF) staining of ZMAT1 and p53 in 60 PDAC tissues. **G** IF staining showed ZMAT1 expression level highly correlated with p53 expression. **H** Kaplan–Meier analysis in PDAC patients grouped according to the expression levels of ZMAT1 and p53 showed that PDAC patients with high ZMAT1/high p53 expression had the longest overall survival among all the groups. **I** A schematic diagram for the role of the ZMAT1-SIRT3-p53 axis in regulation of cell cycle and apoptosis in PDAC. All * *P*-value < 0.05, ** *P*-value < 0.01, *** *P*-value < 0.001. Scale bars: 200 μm. *P*-values were assessed using two-tailed t-tests and ANOVA followed by Dunnett’s tests for multiple comparison in B-C. Spearman’s correlation was performed in G. Kaplan–Meier analyses and log-rank tests were conducted in H

To further evaluate the effects of ZMAT1 on tumor growth in vivo, SW1990 ZMAT1-OV cells and control cells were subcutaneously injected in BALB/c-nude mice (*n* = 6 per group). The established xenograft mouse models suggested that ZMAT1 over-expression significantly inhibited tumor growth (Fig. 7A-B). As shown in Fig. 7C, an obvious decrease in tumor weight was observed at the end of the experimental period in the ZMAT1-OV group compared to the control group. In addition, western blot and IHC assays were used to validate the protein levels of ZMAT1, SIRT3, and p53 (Fig. 7D-E). As expected, SIRT3 and p53 were up-regulated in tumor tissues of ZMAT1-OV mice. In addition, the results of IHC assays also indicated lower levels of Ki-67 and higher Tunel expressions in the ZMAT1-OV group (Fig. 7E). These results indicated that ZMAT1 over-expression suppressed PDAC tumor growth in vivo.

### ZMAT1 correlated with p53 expression in human PDAC

Double-labeled IF staining was used to evaluate tumor tissue samples obtained from 60 PDAC patients to verify the association between ZMAT1 and p53 (Fig. 7F). The results showed a strong positive correlation between these two biomarkers (*R* = 0.47, *P* < 0.001) (Fig. 7G). According to the different expression levels of ZMAT1 and p53, we further stratified patients into three groups: patients with high ZMAT1/high p53 expression (Group 1, *n* = 19); patients with high ZMAT1/low p53 expression or low ZMAT1/high p53 expression (Group 2, *n* = 22), and patients with low ZMAT1/low p53 expression (Group 3, *n* = 19). Kaplan–Meier analysis was used to evaluated the differences in OS among the three groups (Fig. 7H). The results showed Group 1 had the best survival rate while the Group 3 had the worst survival rate, indicating PC patients with low ZMAT1/low p53 expression were a high risk and aggressive cancer group.

## Discussion

Finding novel driver genes regulating PDAC initiation and progression is of great value for identifying potential therapeutic targets. In our study, we demonstrated that ZMAT1 was down-regulated in PDAC and the expression of ZMAT1 was correlated with tumor differentiation, tumor stage, and patient survival, indicating it served as a predictive biomarker for PDAC. Furthermore, in vitro and in vivo experiments showed that the over-expression of ZMAT1 suppressed PDAC cells proliferation, migration, and tumor growth. Taking these together, our study identified ZMAT1 as a tumor suppressor in PDAC.

Mutation or loss of P53 is a critical molecular event leading to PC initiation and progression [10, 22]. Studies have shown p53 regulates cancer cell cycle progression by inducing p21 expression [23]. Similarly, p53 also impaired cancer cell apoptosis by affecting BAX expression [24]. In our study, we determined that P53 was a key downstream factor of ZMAT1 and the effects of ZMAT1 on the cell cycle phase S/G2 arrest and cell apoptosis were dependent on p53. A p53 inhibitor rescued the inhibitory effect of ZMAT1 overexpression on cancer cells viability and proliferation. Of note, ZMAT1 influenced P53 expression in BXPC-3 cell line harboring the P53 mutation and SW1990 and Capan-2 cell lines not presenting any P53 mutations, which indicated that ZMAT1 exerted a general regulatory mechanism on P53. In future studies, it will be interesting to investigate association between ZMAT1 and P53 expression in PDAC patients with or without P53 mutation. In addition, by analyzing the survival data of PDAC cohorts, we found patients with low expression of both ZMAT1 and p53 exhibited the worst survival compared to those with ZMAT1 or high expression of p53. Thus, co-analyzing expression of ZMAT1 and p53 may be beneficial for identifying high-risk and aggressive PC.

ZMAT1 is a member of the C2H2-type zinc finger proteins, which are believed to bind to DNA and act as transcription factors. Indeed, the C2H2-type zinc finger proteins are the largest group among all zinc finger classes. Some binding motifs of C2H2-type zinc finger proteins like ZFP335 were identified [25]. However, direct experimental data are missing supporting whether other particular C2H2-type zinc finger proteins bind to DNA, and their associated biological functions. In our study, we showed ZMAT1 directly bound to DNAs using the ChIP assay. Indeed, ChIP sequencing identified 2567 significant ZMAT1-binding peaks and 1079 putative ZMAT1-binding genes. We identified three ZMAT1 binding sites in the promoter region of SIRT3 and this interaction induced the transcription of SIRT3. These results suggested that ZMAT1 may serve as a transcriptional activator in human cells. It will be of great interest to further investigate the structure of ZMAT1 and explore physical interaction between the corresponding binding domains of ZMAT1 and the corresponding DNA fragments.

Our results showed that ZMAT1-induced p53 expression was dependent on SIRT3. Consistently, other studies also found that SIRT3 up-regulated p53. SIRT3 is a member of the Sirtuin family of class III histone deacetylases and is also considered a tumor suppressor in some solid tumors like hepatocellular carcinoma [26]. In HCC cells, Zhang et al. reported that SIRT3 over-expression up-regulated p53 protein levels by reducing Mdm2-mediated p53 degradation [27]. Xiao et al. revealed that SIRT3 activated p53/p21 signaling and caused apoptosis in the A549 cell line [28]. Taken together with these results and our data, SIRT3 is sufficient to activate P53 in pancreatic cancer cells. Specifically, we illustrate a model that ZMAT1 functions in a p53-dependent manner and SIRT3 is an enhanced target and effector of ZMAT1. ZMAT1 binds to the promoter of SIRT3 and promotes the SIRT3 transcription, which activates p53 signaling pathway and affects pancreatic cancer cell proliferation and apoptosis (Fig. 7I). Furthermore, we found overexpression of ZAMT1 inhibited tumor growth in a xenograft tumor model, along with higher expression of SIRT3 and p53. Future studies may involve developing a treatment strategy of forced expression of ZMAT1 via an adenovirus or mRNA-mediated delivery system in a preclinical PDAC model.

## Conclusion

In conclusion, we identified a novel regulatory model in which ZMAT1 promoted the transcription of the SIRT3 gene, which subsequently up-regulated the expression of p53 and contributed to modulate cell cycle progression and apoptosis in PDAC. Thus, ZMAT1 is a tumor suppressor and a vital prognostic biomarker in PDAC and warrants further study in the future.

## Supplementary Information

Below is the link to the electronic supplementary material.

**Additional file 1:Figure S1.** ZMAT1 expressions in pan-cancer. (A) The analyses of ZMAT1 expression in several cancer types in TCGA+GTEx database. (B) Kaplan-Meier analyses showed patients with low ZMAT1 expression had inferior overall survival in adrenocortical carcinoma (ACC), head and neck squamous cell carcinoma (HNSC), lung adenocarcinoma (LUAD), mesothelioma (MESO) and skin cutaneous melanoma (SKCM). Kaplan-Meier analyses and log-rank tests were performed in B.

**Additional file 2:Figure S2.** The morphology of the used cell lines. The cell morphology of SW1990 (A), BXPC-3 (B) and Capan-2 (C) cells before and after transfection.

**Additional file 3:Figure S3.** Wound-healing assays. Wound-healing assays showed ZMAT1 over-expression reduced the migration in SW1990 cells (A), while ZMAT1 knockdown promoted the migration in BXPC-3 (B) and Capan-2 cells (C). All * P-value <0.05, ** P-value <0.01, *** P-value <0.001. Scale bars: 200 μm. P-values were assessed using two-tailed t-tests and ANOVA followed by Dunnett’s tests for multiple comparison.

**Additional file 4:Figure S4.** Correlations between ZMAT1 with key nodes of cell cycle and apoptosis. (A) Correlations between ZMAT1 with Cyclin Dependent Kinases (CDKs) and cyclins in TCGA data. (B) Correlations between ZMAT1 with apoptosis modulators in TCGA data.

**Additional file 5:Figure S5.** Selection of ZMAT1 downstream effectors. (A) The top 10 biological process terms of Gene Ontology (GO) functional analysis on 1079 ZMAT1-binding genes obtained from ChIP-seq. (B) STRING functional enrichment analysis on 46 overlapping genes. (C) Correlations of expressions of ZMAT1 with the selected 8 TP53-associated genes in GEPIA Database.

**Additional file 6:Figure S6.** SIRT3 is down-regulated and correlates with poor survival in Pancreatic Ductal Adenocarcinoma (PDAC). (A) Down-regulation of SIRT3 was identified in PDAC in Oncomine database (Segara’s dataset, Badea’s dataset, Iacobuzio-Donahue’s dataset and Pei’s dataset). (B) Down-regulation of ZMAT1 was identified in PDAC in three individual GEO datasets (GSE15471, GSE16515 and GSE62165). (C) Kaplan-Meier analyses showed PDAC patients with low expression of SIRT3 had inferior OS and DFS in TCGA cohort. P-values were determined by Non-parametric Mann-Whitney U-test in A-B. Kaplan-Meier analyses and log-rank tests were performed in C.

**Additional file 7:Table S1.** Information on PCR primer oligonucleotide sequences. **Table S2.** Information on siRNA targeted oligonucleotide sequences. **Table S3.** Correlation between ZMAT1 expression with clinicopathological characteristics of PDAC patients. **Table S4.** Univariate and multivariate Cox regression analysis of risk factors associated with overall survival. **Table S5.** Univariate and multivariate Cox regression analysis of risk factors associated with disease-free survival.

## Data Availability

The data that support the findings of this study are available from the corresponding author upon reasonable request.

## Abbreviations

PC: Pancreatic cancer
PDAC: Pancreatic ductal adenocarcinoma
ZMAT1: Zinc finger matrin-type 1
SIRT3: Sirtuin 3
TCGA: The Cancer Genome Atlas
GEO: Gene Expression Omnibus
GEPIA: Gene Expression Profiling Interactive Analysis
GTEx: Genotype-Tissue Expression Project
OS: Overall survival
DFS: Disease-free survival
CA19-9: Carbohydrate antigen 19–9
ChIP: Chromatin immunoprecipitation
GO: Gene Ontology
KEGG: Kyoto Encyclopedia of Genes and Genomes
GSEA: Gene set enrichment analysis
PPI: Protein–protein interaction
SRA: Sequence Read Archive
NCBI: National Center for Biotechnology Information
PFTα: Pifithrin-α
FBS: Fetal bovine serum
RT-qPCR: Real-time quantitative polymerase chain reaction
CCK-8: Cell counting Kit-8
IHC: Immunohistochemistry
IF: Immunofluorescence
KD: Knockdown
OV: Over-expression
DEGs: Differentially expressed genes
