# Supplementary figures and images for: ZMAT1 acts as a tumor suppressor in pancreatic ductal adenocarcinoma by inducing SIRT3/p53 signaling pathway
Source: J Exp Clin Cancer Res. 2022 Apr 7;41:130. doi: 10.1186/s13046-022-02310-8 (PMC8988381; doi:10.1186/s13046-022-02310-8)

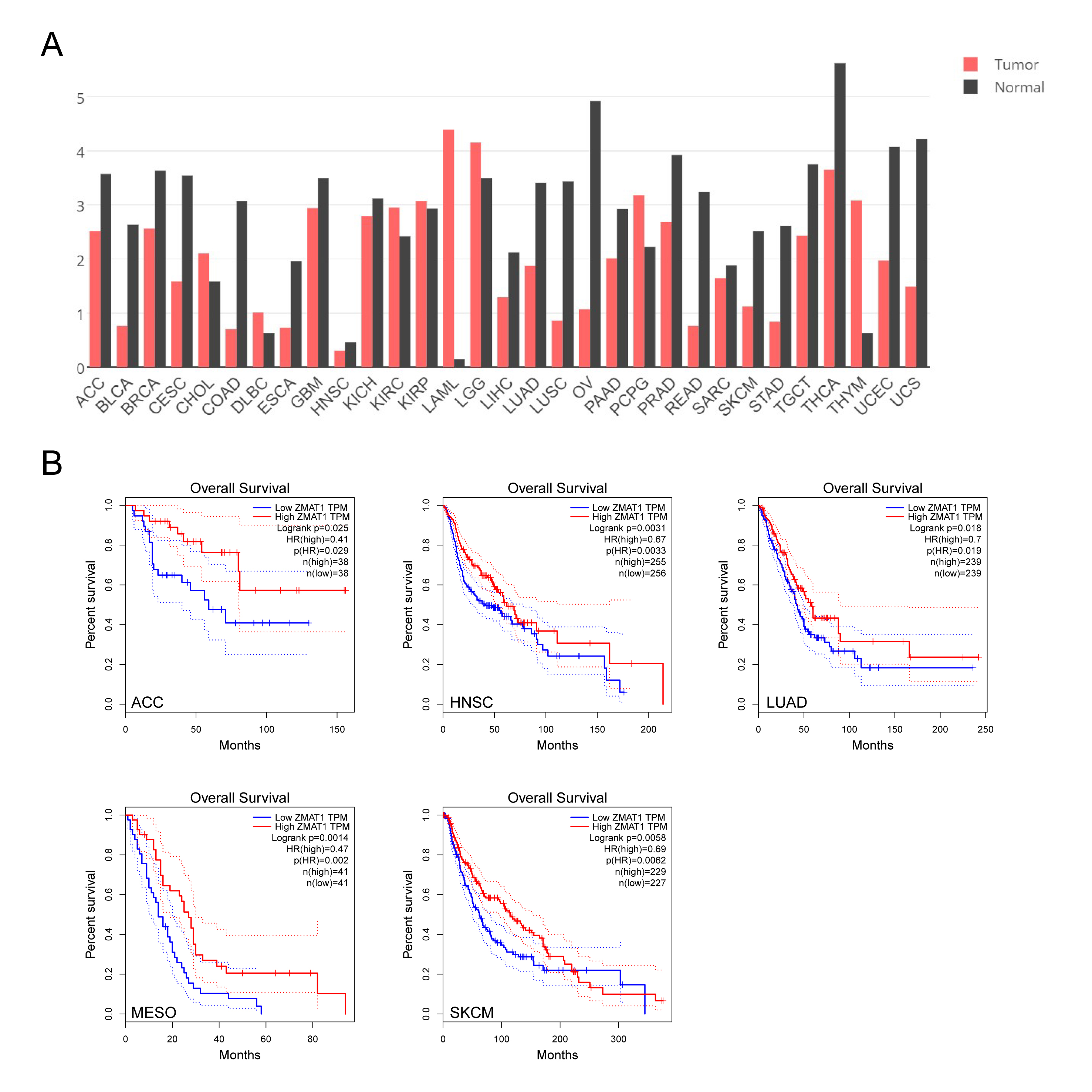

Supplement: Supplementary file 1 — Additional file 1:Figure S1. ZMAT1 expressions in pan-cancer. (A) The analyses of ZMAT1 expression in several cancer types in TCGA+GTEx database. (B) Kaplan-Meier analyses showed patients with low ZMAT1 expression had inferior overall survival in adrenocortical carcinoma (ACC), head and neck squamous cell carcinoma (HNSC), lung adenocarcinoma (LUAD), mesothelioma (MESO) and skin cutaneous melanoma (SKCM). Kaplan-Meier analyses and log-rank tests were performed in B. [file 13046_2022_2310_MOESM1_ESM.jpg]

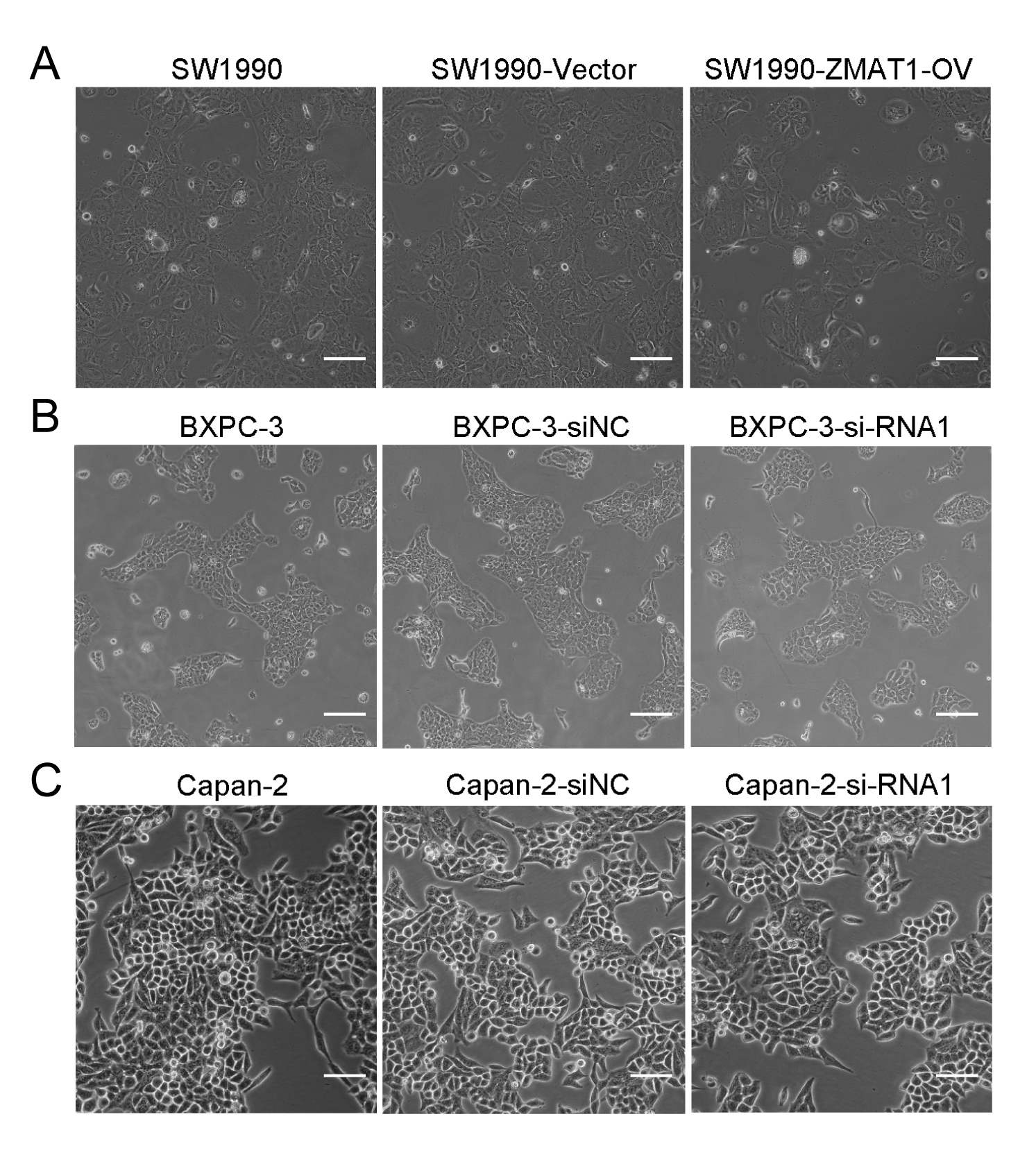

Supplement: Supplementary file 2 — Additional file 2:Figure S2. The morphology of the used cell lines. The cell morphology of SW1990 (A), BXPC-3 (B) and Capan-2 (C) cells before and after transfection. [file 13046_2022_2310_MOESM2_ESM.jpg]

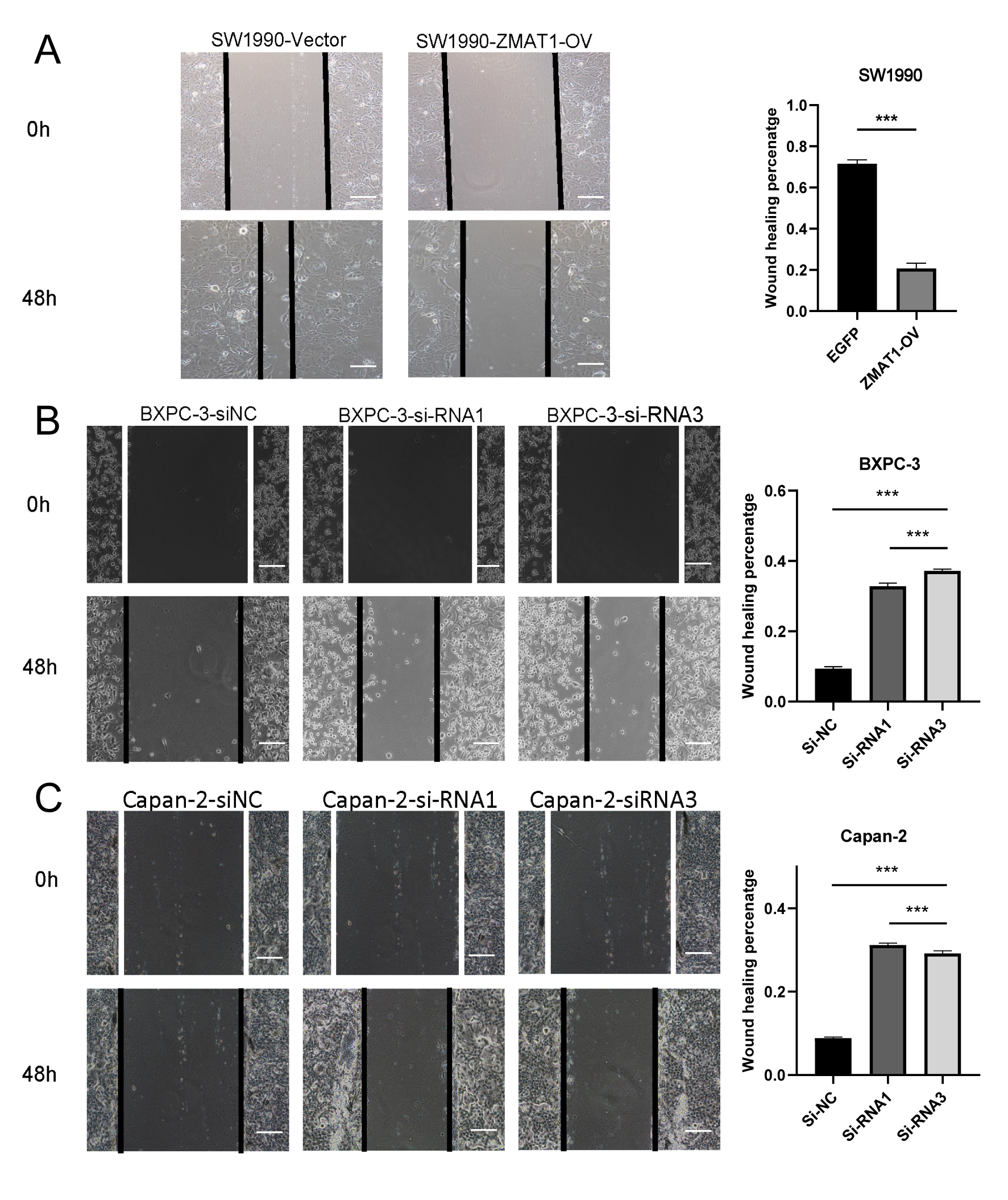

Supplement: Supplementary file 3 — Additional file 3:Figure S3. Wound-healing assays. Wound-healing assays showed ZMAT1 over-expression reduced the migration in SW1990 cells (A), while ZMAT1 knockdown promoted the migration in BXPC-3 (B) and Capan-2 cells (C). All * P-value <0.05, ** P-value <0.01, *** P-value <0.001. Scale bars: 200 μm. P-values were assessed using two-tailed t-tests and ANOVA followed by Dunnett’s tests for multiple comparison. [file 13046_2022_2310_MOESM3_ESM.jpg]

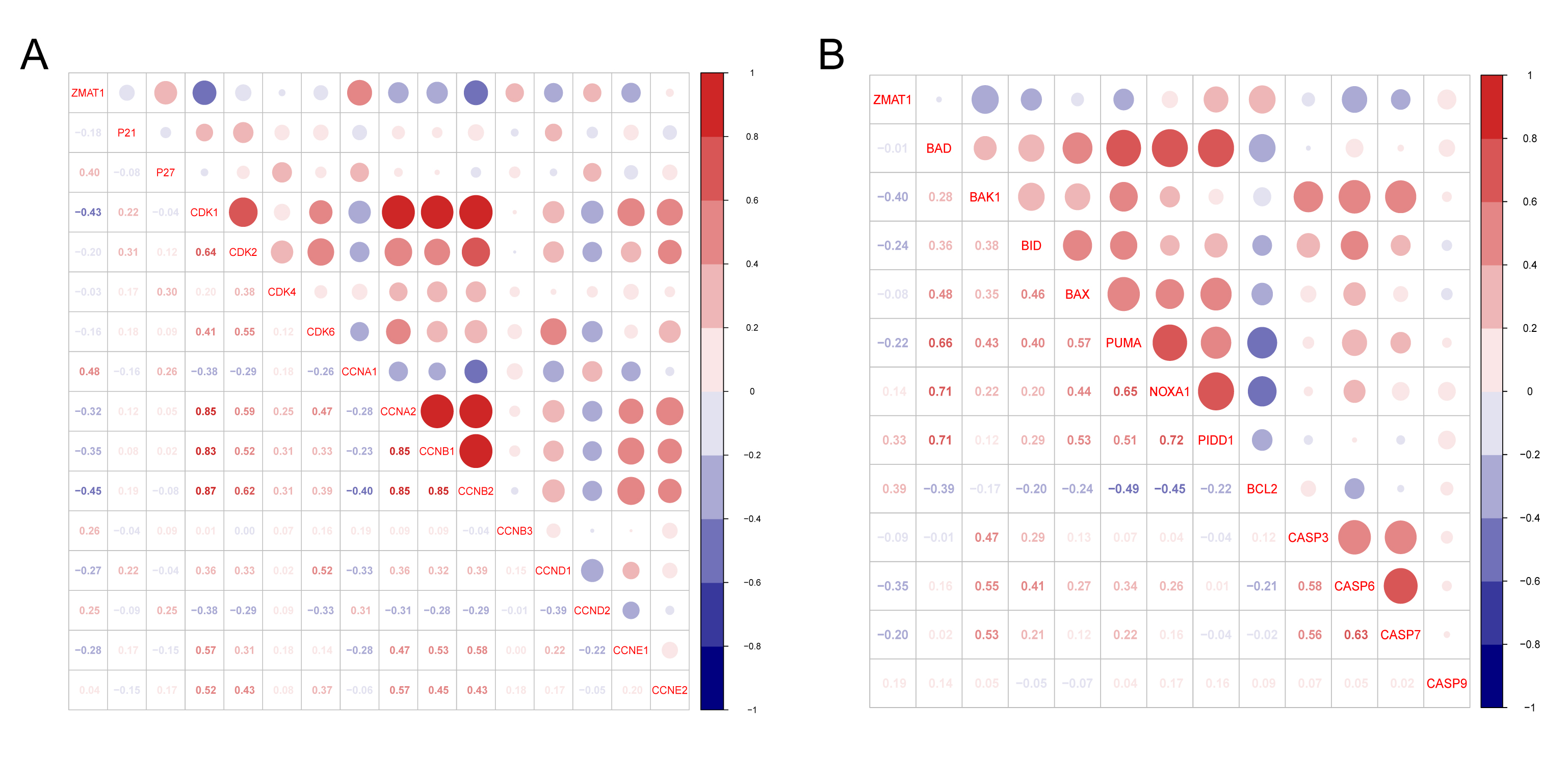

Supplement: Supplementary file 4 — Additional file 4:Figure S4. Correlations between ZMAT1 with key nodes of cell cycle and apoptosis. (A) Correlations between ZMAT1 with Cyclin Dependent Kinases (CDKs) and cyclins in TCGA data. (B) Correlations between ZMAT1 with apoptosis modulators in TCGA data. [file 13046_2022_2310_MOESM4_ESM.jpg]

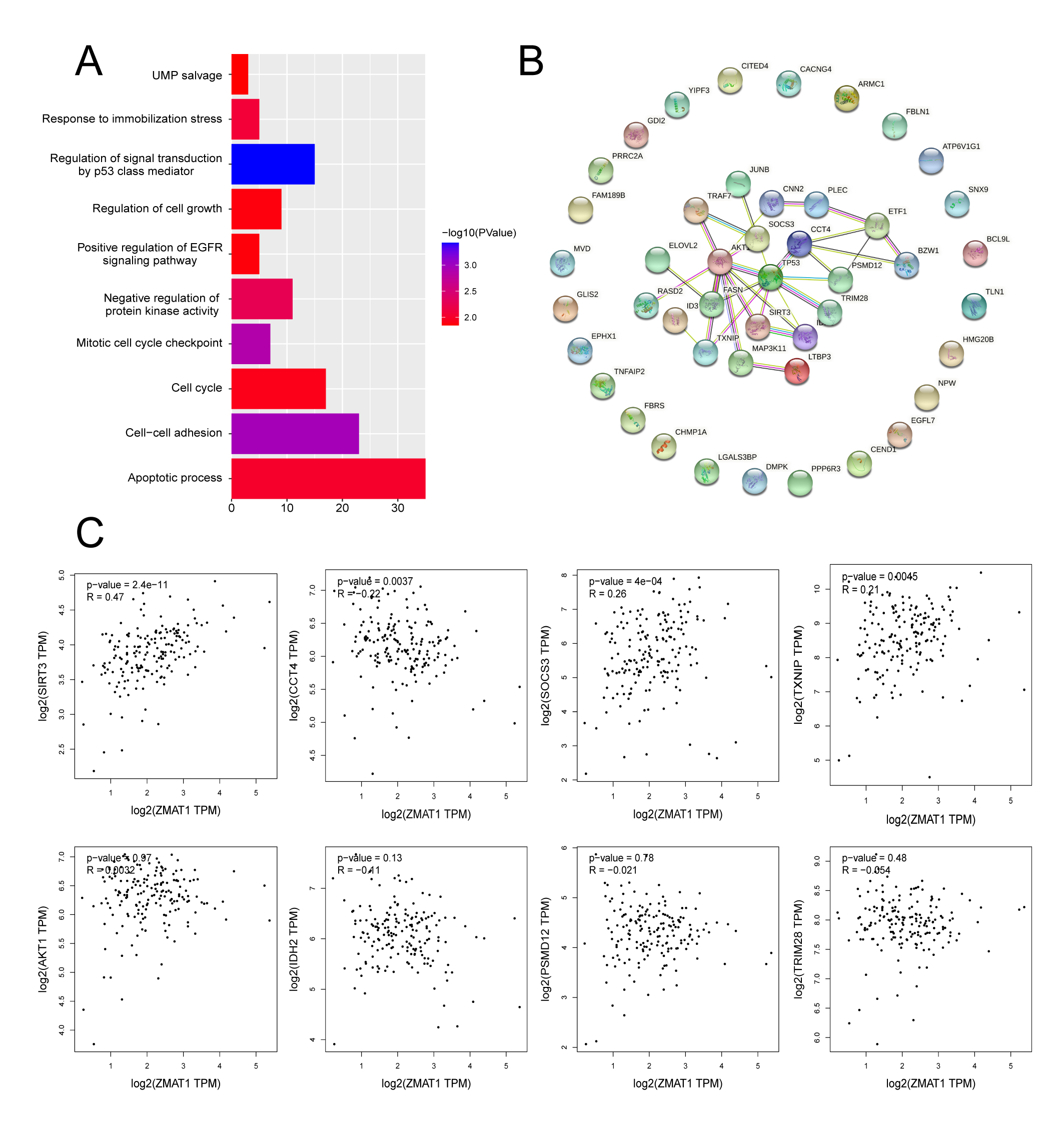

Supplement: Supplementary file 5 — Additional file 5:Figure S5. Selection of ZMAT1 downstream effectors. (A) The top 10 biological process terms of Gene Ontology (GO) functional analysis on 1079 ZMAT1-binding genes obtained from ChIP-seq. (B) STRING functional enrichment analysis on 46 overlapping genes. (C) Correlations of expressions of ZMAT1 with the selected 8 TP53-associated genes in GEPIA Database. [file 13046_2022_2310_MOESM5_ESM.jpg]

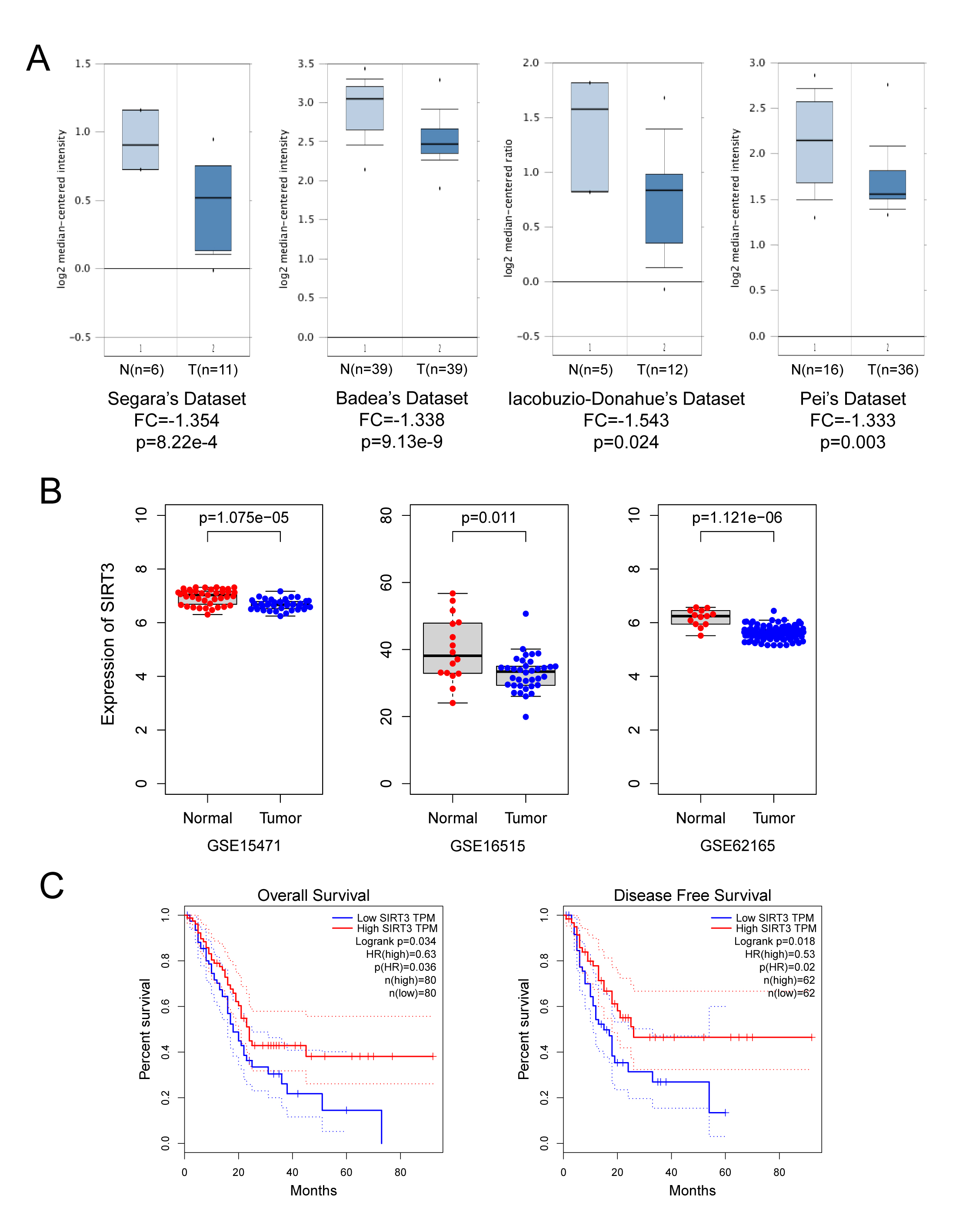

Supplement: Supplementary file 6 — Additional file 6:Figure S6. SIRT3 is down-regulated and correlates with poor survival in Pancreatic Ductal Adenocarcinoma (PDAC). (A) Down-regulation of SIRT3 was identified in PDAC in Oncomine database (Segara’s dataset, Badea’s dataset, Iacobuzio-Donahue’s dataset and Pei’s dataset). (B) Down-regulation of ZMAT1 was identified in PDAC in three individual GEO datasets (GSE15471, GSE16515 and GSE62165). (C) Kaplan-Meier analyses showed PDAC patients with low expression of SIRT3 had inferior OS and DFS in TCGA cohort. P-values were determined by Non-parametric Mann-Whitney U-test in A-B. Kaplan-Meier analyses and log-rank tests were performed in C. [file 13046_2022_2310_MOESM6_ESM.jpg]
