# Supplementary material for: ZMAT1 acts as a tumor suppressor in pancreatic ductal adenocarcinoma by inducing SIRT3/p53 signaling pathway
Source: J Exp Clin Cancer Res. 2022 Apr 7;41:130. doi: 10.1186/s13046-022-02310-8 (PMC8988381; doi:10.1186/s13046-022-02310-8)
Supplement: Supplementary file 7 — Additional file 7:Table S1. Information on PCR primer oligonucleotide sequences. Table S2. Information on siRNA targeted oligonucleotide sequences. Table S3. Correlation between ZMAT1 expression with clinicopathological characteristics of PDAC patients. Table S4. Univariate and multivariate Cox regression analysis of risk factors associated with overall survival. Table S5. Univariate and multivariate Cox regression analysis of risk factors associated with disease-free survival. [file 13046_2022_2310_MOESM7_ESM.docx]

**Table S1. Information on PCR primer oligonucleotide sequences.**

| **ID** | **Sequence (5’- 3’)** | **Location** | **Length (bp)** | **Usage** |
| --- | --- | --- | --- | --- |
| ZMAT1 F | TGTGGAGTGATGCTACAGTTTG | 181-202 | 114 | qPCR |
| ZMAT1 R | AGGCACTTCATTTTGTTCCCC | 294-274 |  | qPCR |
| P53 F | CAGCACATGACGGAGGTTGT | 382-401 | 125 | qPCR |
| P53 R | TCATCCAAATACTCCACACGC | 506-486 |  | qPCR |
| P21 F | TGTCCGTCAGAACCCATGC | 21-39 | 139 | qPCR |
| P21 R | AAAGTCGAAGTTCCATCGCTC | 159-139 |  | qPCR |
| BAD F | CCCAGAGTTTGAGCCGAGTG | 12-31 | 249 | qPCR |
| BAD R | CCCATCCCTTCGTCGTCCT | 260-242 |  | qPCR |
| SIRT3 F | ACCCAGTGGCATTCCAGAC | 24-42 | 123 | qPCR |
| SIRT3 R | GGCTTGGGGTTGTGAAAGAAG | 146-126 |  | qPCR |
| TXNIP F | GGTCTTTAACGACCCTGAAAAGG | 36-58 | 87 | qPCR |
| TXNIP R | ACACGAGTAACTTCACACACCT | 122-101 |  | qPCR |
| CCT4 F | ATGCCCGAGAATGTGGCAC | 1-19 | 88 | qPCR |
| CCT4 R | GCTTGTCGCGGTCCTGATAG | 88-69 |  | qPCR |
| SOCS3 F | CCTGCGCCTCAAGACCTTC | 57-75 | 99 | qPCR |
| SOCS3 R | GTCACTGCGCTCCAGTAGAA | 155-136 |  | qPCR |
| GAPDH F | GGAGCGAGATCCCTCCAAAAT | 108-128 | 197 | qPCR |
| GAPDH R | GGCTGTTGTCATACTTCTCATGG | 304-282 |  | qPCR |
| SIRT3-1 F | CAGGTTACTACTTGGAGAA |  |  | ChIP-qPCR |
| SIRT3-1 R | GTTGGATAGCACTTGGTA |  |  | ChIP-qPCR |
| SIRT3-2 F | GGAGGTTACTACAGGAGATG |  |  | ChIP-qPCR |
| SIRT3-2 R | GCCGTCCCATTGTCTTTA |  |  | ChIP-qPCR |

**Table S2. Information on siRNA targeted oligonucleotide sequences.**

| **Symbol** | **PrimerName** | **Sequence (5’- 3’)** | **Length (bp)** |
| --- | --- | --- | --- |
| ZMAT1 | ZMAT1(human) siRNA-1 | GCAAUUAAUGGAGGAACAUTT | 21 |
|  | ZMAT1(human) siRNA-2 | GCAAGGAAGUGAACAUCAATT | 21 |
|  | ZMAT1(human) siRNA-3 | GCAGAAAGCCAGAGGACUATT | 21 |
| SIRT3 | SIRT3(human) siRNA-1 | GAAUGAAGUUAGUUUCUAATT | 21 |
|  | SIRT3(human) siRNA-2 | GCUUGAUGGACCAGACAAATT | 21 |
|  | SIRT3(human) siRNA-3 | GCCUGUUUGCUGUGUUGAATT | 21 |

**Table S3. Correlation between ZMAT1 expression with clinicopathological characteristics of PDAC patients.**

| **Clinicopathological variables** | **Patients (n=122)** | **ZMAT1 expression** | | **P-Value** |
| --- | --- | --- | --- | --- |
|  |  | **High (56)** | **Low (66)** |  |
| Gender |  |  |  |  |
| Male | 65 | 33 | 32 | 0.332 |
| Female | 57 | 23 | 34 |  |
| Age |  |  |  |  |
| ≧60 | 70 | 29 | 41 | 0.332 |
| <60 | 52 | 27 | 25 |  |
| CA19-9 |  |  |  |  |
| ≧300 ng/ml | 68 | 24 | 44 | **<0.05** |
| <300 ng/ml | 54 | 32 | 22 |  |
| Lymphnodes metastasis |  |  |  |  |
| Positive | 42 | 13 | 29 | **<0.05** |
| Negative | 80 | 43 | 37 |  |
| Perineural invasion |  |  |  |  |
| Present | 34 | 15 | 19 | 0.966 |
| Absent | 88 | 41 | 47 |  |
| TNM stage |  |  |  |  |
| Advanced (III & IV) | 46 | 11 | 35 | **<0.01** |
| Early (I & II) | 76 | 45 | 31 |  |
| Differentiation |  |  |  |  |
| Poor | 57 | 20 | 37 | **<0.05** |
| Well | 65 | 36 | 29 |  |

**Table S4. Univariate and multivariate Cox regression analysis of risk factors associated with overall survival.**

| **Clinicopathological variables** | **Univariate analysis** | | | **Multivariate analysis** | | |
| --- | --- | --- | --- | --- | --- | --- |
|  | **HR** | **95% CI** | **P-Value** | **HR** | **95% CI** | **P-Value** |
| ZMAT1 expression (Low vs. High) | 6.82 | 5.41-8.23 | **<0.01** | 4.26 | 3.39-5.13 | **<0.01** |
| Gender (Male vs. Female) | 1.92 | 0.83-3.01 | 0.33 |  |  |  |
| Age (≧60 vs. <60) | 1.21 | 0.53-1.89 | 0.62 |  |  |  |
| CA19-9 (≧300 ng/ml vs. <300 ng/ml) | 1.79 | 0.92-2.66 | 0.08 |  |  |  |
| Lymphnodes metastasis (Positive vs. Negative) | 1.96 | 1.18-2.74 | **<0.05** | 1.70 | 0.46-2.94 | 0.32 |
| Perineural invasion (Present vs. Absent) | 2.97 | 2.22-3.72 | **<0.01** | 2.94 | 1.69-4.19 | **<0.05** |
| TNM stage (Advanced vs. Early) | 3.84 | 3.08-4.60 | **<0.01** | 3.54 | 1.96-5.11 | **<0.01** |
| Differentiation (Poor vs. Well) | 2.24 | 1.36-3.12 | **<0.05** | 1.92 | 1.15-2.69 | **<0.05** |

**Table S5. Univariate and multivariate Cox regression analysis of risk factors associated with disease-free survival.**

| **Clinicopathological variables** | **Univariate analysis** | | | **Multivariate analysis** | | |
| --- | --- | --- | --- | --- | --- | --- |
|  | **HR** | **95% CI** | **P-Value** | **HR** | **95% CI** | **P-Value** |
| ZMAT1 expression (Low vs. High) | 5.71 | 4.27-7.15 | **<0.01** | 3.41 | 2.11-4.71 | **<0.01** |
| Gender (Male vs. Female) | 1.32 | 0.79-1.85 | 0.45 |  |  |  |
| Age (≧60 vs. <60) | 1.12 | 0.41-1.83 | 0.63 |  |  |  |
| CA19-9 (≧300 ng/ml vs. <300 ng/ml) | 1.42 | 0.89-1.95 | 0.13 |  |  |  |
| Lymphnodes metastasis (Positive vs. Negative) | 2.20 | 1.45-2.95 | **<0.05** | 1.45 | 0.29-2.61 | 0.63 |
| Perineural invasion (Present vs. Absent) | 3.45 | 2.25-4.65 | **<0.01** | 2.21 | 1.27-3.15 | **<0.05** |
| TNM stage (Advanced vs. Early) | 3.91 | 2.99-4.83 | **<0.01** | 2.92 | 1.79-4.05 | **<0.05** |
| Differentiation (Poor vs. Well) | 2.37 | 1.41-3.33 | **<0.05** | 1.63 | 0.85-2.41 | 0.34 |
